# Prenatal exposure to phthalates and autism spectrum disorder in the MARBLES study

**DOI:** 10.1186/s12940-018-0428-4

**Published:** 2018-12-05

**Authors:** Hyeong-Moo Shin, Rebecca J. Schmidt, Daniel Tancredi, Jacqueline Barkoski, Sally Ozonoff, Deborah H. Bennett, Irva Hertz-Picciotto

**Affiliations:** 1 Department of Public Health Sciences, University of California, Davis, California USA; 2 Department of Earth and Environmental Sciences, University of Texas, Arlington, TX USA; 3 Department of Pediatrics, University of California, Davis, California USA; 4 UC Davis MIND (Medical Investigations of Neurodevelopmental Disorders) Institute, Sacramento, California USA; 5 Department of Psychiatry and Behavioral Sciences, University of California Davis Medical Center, Sacramento, California USA

**Keywords:** Autism spectrum disorder, Exposure, Phthalates, Pregnancy, Urine samples

## Abstract

**Background:**

Evidence from experimental and observational studies suggests that prenatal phthalate exposures may be associated with autism spectrum disorder (ASD). We examined whether prenatal phthalate exposures were associated with an increased risk of ASD.

**Methods:**

We quantified 14 metabolites of eight phthalates in 636 multiple maternal urine samples collected during 2nd and 3rd trimesters of pregnancy from 201 mother-child pairs in MARBLES (*M*arkers of *A*utism *R*isk in *B*abies – *L*earning *E*arly *S*igns), a high-risk ASD longitudinal cohort. At 3 years old, children were clinically assessed for ASD and classified into three diagnostic categories: ASD (*n* = 46), non-typical development (Non-TD, *n* = 55), and typical development (TD, *n* = 100). We used multinomial logistic regression to evaluate the association of phthalate metabolite concentrations with ASD and Non-TD.

**Results:**

Most associations of phthalate biomarkers with both ASD and Non-TD were null, with the exception that monoethyl phthalate (MEP) was significantly associated with an increased risk of Non-TD (per 2.72-fold relative increase in concentration: Relative risk ratio (RRR) = 1.38; 95% confidence interval (CI): 1.01, 1.90). When stratified by prenatal vitamin use during the first month of pregnancy, among mothers who took vitamins, ASD risk was inversely associated with mono-isobutyl phthalate (MiBP, RRR = 0.44; 95% CI: 0.21, 0.88), mono(3-carboxypropyl) phthalate (MCPP, RRR = 0.41; 95% CI: 0.20, 0.83) and mono-carboxyisooctyl phthalate (MCOP, RRR = 0.49; 95% CI: 0.27, 0.88), but among mothers who did *not* take prenatal vitamins, Non-TD risk was positively associated with MCPP (RRR = 5.09; 95% CI: 2.05, 12.6), MCOP (RRR = 1.86; 95% CI: 1.01, 3.39), and mono-carboxyisononyl phthalate (MCNP, RRR = 3.67; 95% CI: 1.80, 7.48). When stratified by sex, among boys, MEP, monobenzyl phthalate, MCPP, MCNP, and sum of di(2-ethylhexyl) phthalate metabolites (ΣDEHP) were positively associated with Non-TD risk, but associations with ASD were null. Among girls, associations with both ASD and Non-TD were null.

**Conclusions:**

Our study showed that phthalate exposures in mid- to late pregnancy were not associated with ASD in children from this high-risk ASD cohort. Further studies should be conducted in the general population without high-risk genes to confirm our findings.

**Electronic supplementary material:**

The online version of this article (10.1186/s12940-018-0428-4) contains supplementary material, which is available to authorized users.

## Background

Autism spectrum disorder (ASD) is of considerable public health importance and little is known about non-genetic causes of ASD [1]. A recent study reported that ASD affects about 1 in 59 children in the United States [1]. Evidence about overall environmental risk factors of ASD is growing, but studies about the contribution of endocrine disrupting chemicals (EDCs) to the etiology of ASD are still limited [2]. Among EDCs, phthalates are suspected to contribute to ASD risk [3]. Phthalates are widely used in personal care products such as cosmetics, fragrances, and shampoos, and in indoor residential environments such as polyvinyl chloride (PVC) flooring and plastics, children’s toys, vinyl tiles, and shower curtains [4–6]. Because of widespread use of phthalates, phthalate biomarkers have been detected in the urine of the majority of the U.S. general population [7]. Phthalate biomarkers have also been detected in the urine of pregnant women [8–15]. Because rat studies showed that phthalates cross the placenta to the fetus [16], the possibility of gestational exposures during critical periods of development exists.

Phthalates have toxic effects potentially relevant to ASD etiology. Rat studies showed that phthalates have neuro-developmental toxicities and were also associated with neuro-behavioral outcomes [17–23]. Some phthalates are known to affect thyroid hormone homeostasis in pregnant women [24–30]. Maintaining maternal thyroid homeostasis during pregnancy is known to be important for fetal growth and development, particularly fetal neurodevelopment [31, 32]. Thus, it is hypothesized that prenatal exposure to phthalates might contribute to risk of childhood neurodevelopmental disorders. Among children 8–11 years of age, urinary concentrations of di(2-ethylhexyl) phthalate (DEHP) metabolites were significantly associated with attention deficit hyperactivity disorder (ADHD) [33, 34]. Other studies also found that having PVC flooring (a source of certain phthalates) in a parent’s bedroom during pregnancy and child’s first year was associated with an increased risk of ASD [35] and that higher indoor dust concentrations of diethyl phthalate (DEP) and dibutyl phthalate (DBP) were associated with greater hyperactivity-impulsivity and inattention among ASD children [36].

Many environmental epidemiologic studies of ASD have relied on either retrospective self-reports of exposures, or biological markers collected after the time of diagnosis and lacked measurements from the critical time windows of exposure such as pregnancy or early infancy. Because ASD is relatively rare [1], the most efficient way to evaluate its relationship to exposures is through a large case-control study, which by definition, requires retrospective exposure assessment. However, use of enriched-risk cohorts offers the advantage of utilizing biological samples collected prospectively with an efficient design that capitalizes on younger siblings who are at high risk for developmental concerns [37].

For the present study, we used multiple maternal pregnancy urine samples collected in the MARBLES (*M*arkers of *A*utism *R*isk in *B*abies – *L*earning *E*arly *S*igns) study that enrolls pregnant women carrying fetuses at high risk for later developing ASD. This present study improves upon the methodologies of previous studies through clinical confirmation of neurodevelopmental outcomes by trained clinical psychologists with demonstrated reliability, and a strong exposure assessment strategy based on multiple biomarker measurements in multiple trimesters of pregnancy. The goal of this study was to determine whether exposure to phthalates during mid- to late pregnancy was associated with an increased risk of ASD in this high-risk ASD longitudinal cohort.

## Methods

### Study population

Participants were from the MARBLES study that follows pregnant women who are at high risk (~ 1 in 5) for delivering another infant(s) who will develop ASD, primarily because they previously delivered a child who developed ASD [38]. Two women did not have a child who developed ASD before enrollment but were included in MARBLES because they were at high risk of having a child with ASD (e.g., mother had an identical twin with an ASD child). Mothers included in this study were recruited between 2006 and 2014, mostly in Northern California. Although all of these offspring are at high risk, only some will develop ASD, others will have different developmental concerns, and many will develop typically [39]. MARBLES families are primarily recruited from lists of children receiving services for autism through the California Department of Developmental Services, as well as from other studies, by self- or provider referrals and obstetrics/gynecology clinics. Details of study design, recruitment, eligibility, sample size, exposure data, and developmental diagnosis are available elsewhere [40].

For the present study, we selected 186 mothers who (1) provided first morning voids (FMV) and/or 24-h urine samples during pregnancy collected from 01/2007 to 02/2014 and (2) had a child who completed the study at 3 years of age. Among 186 mothers, 10 mothers participated in the study for two pregnancies and one mother participated for three pregnancies over 4 years. All urine samples included in the present study were collected from a total of 198 unique pregnancies. A total of 201 children were included in the present study because three pregnancies resulted in twins (see Additional file 1: Figure S1 for the selection of 201 mother-child pairs used in the current study).

### Child neurodevelopmental assessment

Children at 3 years old were assessed for ASD by licensed clinical psychologists using the gold standard Autism Diagnostic Observation Schedules (ADOS) [41]. Children were also administered the Mullen Scales of Early Learning (MSEL), a standardized instrument for ages 3 to 60 months to assess cognitive development [42, 43]. The MSEL generates five subscale scores (gross motor, fine motor, expressive language, receptive language, and visual reception). A previously published algorithm was used to classify neurodevelopmental outcomes into categories using ADOS scores and MSEL scores [44, 45]. Participants with ASD outcomes (*n* = 46) had scores over the ADOS cutoff and met the Diagnostic and Statistical Manual of Mental Disorders (DSM) criteria for ASD. Participants with non-typical development outcomes (Non-TD, *n* = 55) had scores within three points of the ADOS cutoff and/or Mullen scores 1.5 to 2 standard deviations below average. The rest of the sample were classified with typical development outcomes (TD, *n* = 100). Details of selection criteria for each categorization are available elsewhere [44].

### Urine sample collection

Each woman in the MARBLES study was instructed to collect three FMVs (taken 1 week apart) and one 24-h urine sample in each trimester, placing samples collected prior to the day of her visit in their home refrigerator or freezer. Samples were returned to the laboratory at UC Davis, thawed, aliquoted, and then stored at − 80 °C at the UC Davis biorepository. Details of collection methods for FMVs and 24-h samples are available elsewhere [46]. Although almost all of the mothers included in the current study provided urine samples during the 2nd and 3rd trimesters, only 72 mothers provided urine samples during the 1st trimester and most of them provided a single urine sample. Thus, a total of 1020 urine samples (774 FMV and 246 24-h samples) collected during the 2nd and 3rd trimesters were used in the current study.

To reduce analytical cost, for mothers who provided three or more urine specimens within a trimester, we selected the first FMV as an individual sample and pooled all remaining samples for that trimester. To pool urine specimens, 5 mL of each specimen was transferred by sterile pipette into a sterile 20 mL conical tube (or 50 mL conical tube if more than 4 specimens were pooled) and combined using a vortex mixer for 10 s. Next, 2 mL aliquots of the pooled specimen were transferred by pipette into sterile vials [46]. When no FMV was available but a 24-h sample was the only sample obtained within a given trimester, we selected those 24-h samples in lieu of an FMV. The 24-h samples comprised 18.8% of the 437 individual samples analyzed in this study. After pooling, a total of 636 urine samples remained for analysis: 437 individual samples (355 FMV samples and 82 24-h samples) and 199 within-subject pools (28.1% of the pools were comprised of 24-h samples). The number of individual samples included in each of the 199 pools varied based on the number of samples each woman collected. The type and number of urine samples collected and analyzed in this study are summarized (see Additional file 1: Figure S2). The number of individual and pooled samples used to characterize average exposure varied by mother, because the number of samples each woman collected for each trimester varied. All ten possible combinations of selected individual and pooled samples including two exceptions are summarized (see Additional file 1: Table S1).

### Urinary phthalate metabolite quantification

We shipped the urine samples in 2 mL aliquots to the Centers for Disease Control and Prevention (CDC) for analysis. At CDC, we quantified the urinary concentrations for 14 metabolites of eight phthalates using online solid phase extraction coupled with high-performance liquid chromatography with isotope dilution-tandem mass spectrometry as described elsewhere [47]. The 14 metabolites quantified included: monoethyl phthalate (MEP), mono-isobutyl phthalate (MiBP), monohydroxy-isobutyl phthalate (MHiBP), mono-n-butyl phthalate (MBP), monohydroxy-n-butyl phthalate (MHBP), monobenzyl phthalate (MBzP), mono(2-ethylhexyl) phthalate (MEHP), mono(2-ethyl-5-hydroxyhexyl) phthalate (MEHHP), mono(2-ethyl-5-oxohexyl) phthalate (MEOHP), mono(2-ethyl-5-carboxypentyl) phthalate (MECPP), mono(3-carboxypropyl) phthalate (MCPP), mono-isononyl phthalate (MNP), mono-carboxyisooctyl phthalate (MCOP), and mono-carboxyisononyl phthalate (MCNP). In addition to study samples, the laboratory analyzed 51 duplicates for quality assurance. Replicate analyses for individual pairs of duplicate samples showed good agreement: the average relative percent difference was 7% (range: 4–15%, depending on the analyte).

### Exposure metric

For concentrations below the limit of detection (LOD), we used machine-observed concentrations without substituting values to minimize bias associated with such practices [48, 49]. The LODs varied between 0.2 and 1.2 micrograms per liter (μg/L), depending on the analyte. To account for urinary dilution [9, 50], we measured specific gravity (SG) in each sample (either individual or pooled) using a digital handheld refractometer (Atago Co., Ltd., Tokyo, Japan) at UC Davis. SG for all samples included in this study ranged from 1.001 to 1.033, and summary statistics for SG of all samples, FMV, 24-h samples, and pools are provided in the (see Additional file 1: Table S2). Urinary phthalate metabolite concentrations were corrected for dilution using the following formula: C_SG_ = C [(1.012–1)/(SG – 1)], where C_SG_ is the SG-corrected metabolite concentration (in μg/L), C is the measured metabolite concentration in urine (in μg/L), 1.012 is the median SG of all samples analyzed, and SG is the specific gravity of each sample [14]. The SG-corrected concentrations with a correction factor, a computed value in the bracket of the above formula, might not be reliable if the factor is outside the range of 0.5 and 2.0 [51]. Approximately 90% of the samples had an SG-correction factor within this range; however we trimmed correction factors below this range to 0.5 (*n* = 24) and correction factors above this range to 2.0 (*n* = 53).

To account for the varying number of urine samples per mother used for analysis, we calculated a weighted average of phthalate metabolite concentration (C_ave_) during mid- to late pregnancy, with the weights proportional to the number of individual and pooled specimens used to form the concentration. For example, when phthalate concentrations for one FMV and one pooled sample with three composites were only available for one of the mothers, then a weighted average phthalate metabolite concentration for the mother was calculated using the following formula: C_ave_ = (C_ind_ + C_pooled_ × N_pooled_)/(N_ind_ + N_pooled_), where C_ind_ is the phthalate metabolite concentration in the individual sample, C_pooled_ is the phthalate metabolite concentration in the pooled sample, N_pooled_ is the number of composites in the pooled sample, and N_ind_ is the number of the individual sample (=1). Metabolite concentrations in our samples showed a skewed distribution so we used the mean of the ln-transformed SG-corrected concentrations as an average of the pregnancy exposure. Due to high correlations among four DEHP metabolites (*r* = 0.84 to 0.99, see Additional file 1: Table S3), we used the molar sum of DEHP metabolites (ΣDEHP = MEHP + MEHHP + MEOHP + MECPP; μmol/L) in our analysis, instead of individual DEHP metabolites. Lastly, we computed the average phthalate biomarker concentration for each of the 2nd and 3rd trimesters to investigate trimester-specific effects of phthalate exposures on ASD.

### Statistical analysis

We fit multinomial logistic regression models to examine the association between prenatal exposure to phthalates and the risk of ASD or Non-TD in our children. Confounders were selected a priori based on a directed acyclic graph (DAG) and the risk factors of ASD obtained from literature review (see Additional file 1: Figure S3 for DAG). Covariates adjusted in the final model included child’s birth year (continuous), maternal pre-pregnancy body mass index (BMI; underweight/normal weight, overweight, obese), and homeownership (owner, non-owner) as a proxy of socioeconomic status. Child’s birth year was adjusted, because some of the phthalate biomarker concentrations were increasing or decreasing over our sample collection period (see Additional file 1: Figure S4), as also observed within the U.S. general population [52], and the rate of ASD diagnosis was also changing over the period (Table 1). To robustly control confounding by child’s birth year, we centered birth year at 2010 and then included both linear and squared terms in the model. For mothers who participated in the study for multiple pregnancies (*n* = 11), we clustered them by participant identification numbers in the regression models by using variance estimators (‘vce’) options in STATA (https://www.stata.com/manuals13/xtvce_options.pdf) because some of the covariates (e.g., maternal pre-pregnancy BMI, homeownership) of these mothers are not independent between pregnancies.

**Table 1:** Characteristics of participants (*n* = 186 mothers, *n* = 201 children) included in the present study

Characteristics	Row total	TD	ASD	Non-TD	TD	ASD	Non-TD	ASD versus TD ^a^	Non-TD versus TD ^a^
	*n*	*n*			%				
Child’s sex								0.04	0.25
Girl	79	46	13	20	46	28	36		
Boy	122	54	33	35	54	72	64		
Birth year								0.04	0.21
2007	11	7	1	3	7	2	5		
2008	35	26	3	8	26	7	15		
2009	46	23	11	13	23	24	24		
2010	31	15	8	8	15	17	15		
2011	19	9	6	4	9	13	7		
2012	17	7	8	2	7	17	4		
2013	34	10	9	15	10	20	27		
2014	5	3	0	2	3	0	4		
Child’s race/ethnicity								0.19	0.07
White (non-Hispanic)	195	95	45	55	53	37	35		
Hispanic	6	5	1	0	26	37	31		
Other ^b^	195	95	45	55	21	26	35		
Birth Status								0.42	0.09
Singleton	195	95	45	55	95	98	100		
Has a twin	6	5	1	0	5	2	0		
Gestational age at delivery								0.10	0.14
≤ 37 weeks	18	13	2	3	13	4	6		
> 37 weeks	180	85	44	51	87	96	94		
Maternal pre-pregnancy BMI								0.21	0.41
Normal/ underweight	103	57	20	26	58	43	48		
Overweight	48	22	13	13	22	28	24		
Obese	46	19	12	15	19	26	28		
Missing	1	0	1	0	0	2	0		
Maximum parental education								0.56	0.43
Less than college degree	62	29	15	18	28	36	38		
Bachelor’s degree	87	49	20	18	48	48	38		
Graduate or professional degree	43	24	7	12	24	17	25		
Mother’s age at delivery								0.13	0.68
< 35 years	105	56	20	29	57	43	54		
≥ 35 years	93	42	26	25	43	57	46		
Father’s age at delivery								0.18	0.51
< 35 years	75	38	12	21	39	27	40		
≥ 35 years	118	58	31	30	60	69	57		
Missing	5	1	2	2	1	4	4		
Prenatal vitamin use in the first month of pregnancy								0.00	0.37
Yes	105	61	15	29	62	33	54		
No	85	36	26	23	37	57	43		
Missing	8	1	5	2	1	11	4		
Homeowner								0.07	0.37
Yes	117	64	23	30	65	50	56		
No	75	33	20	22	34	43	41		
Missing	6	1	3	2	1	7	4		
Parity								0.08	0.32
0 ^c^	2	2	0	0	2	0	0		
1	80	44	14	22	45	30	41		
> 1	110	51	29	30	52	63	56		
Missing	6	1	3	2	1	7	4		
Mom ever smoked								0.13	0.46
Yes	70	31	20	19	32	43	35		
No	123	66	24	33	67	52	61		
Missing	5	1	2	2	1	4	4		
Season of conception (months)								0.29	0.30
Cold (November – April)	100	54	21	25	55	46	46		
Warm (May – October)	98	44	25	29	45	54	54		
Number of urine samples collected								0.60	0.09
1–2	35	17	9	8	17	20	15		
3–4	54	23	16	16	23	35	30		
5–6	37	24	8	7	24	17	13		
7–8	57	30	11	15	31	24	28		
9–12	15	4	2	8	4	4	15		

^a^P-value from the Pearson’s chi-squared test

^b^Includes Black, Asian, and multiracial

^c^One of them has an identical twin sister with an autistic child, and the other mother has multiple siblings with autism. These two mothers were included in the current study because they have high-risk ASD genetic factors and exclusion of them did not change the results

Note: Eleven mothers participated in the study for two different pregnancies and one mother participated for three different pregnancies over 4 years. Three pregnancies resulted in twins

Based on previous work describing biologic plausibility and evidence for interactions between neurodevelopmental outcomes and other contaminants by sex and nutrient supplements [53–59], we examined child’s sex and self-reported maternal prenatal vitamin use during the first month of pregnancy as effect modifiers in stratified analyses and by adding interaction product terms between the phthalate exposure metric and the potential effect modifier (sex or prenatal vitamins) in the separate regression models. Prenatal vitamin use for each month from 6 months before until the end of pregnancy (and during breast feeding) was prospectively collected through maternal interviews; for this analysis we focused on the first month of pregnancy, given this was the month with the strongest inverse association with ASD [60]. As sensitivity analyses, we ran a series of additional models, further adjusting for (1) interpregnancy interval (continuous), (2) gestational age at birth (continuous), (3) maternal age at delivery (continuous), and (4) child’s race/ethnicity (white, Hispanic, other). As separate analyses, we excluded the top 2.5 percentiles of biomarker concentrations to examine the effect due to extreme values, and restricted our analysis to singleton births by excluding 3 pairs of twins. We also applied analytical weights to the regression models to examine the effect due to the varying number of urine samples per mother, using the methods available elsewhere [61, 62].

All statistical analyses were performed using STATA/MP version 15.1 (StataCorp LLC, College Station, TX, USA), SAS version 9.4 (Institute Inc. Carny, NC, USA), and MATLAB 2018a (Mathworks, Inc., Natick, MA, USA).

## Results

### Population characteristics

In this study, the male to female ratio was 1.2, 2.5, and 1.8 among TD, ASD, and Non-TD, respectively (Table 1). TD children tended to be born in earlier study years than ASD children (*p*-value for Pearson’s chi-squared test was 0.04). Among TD children, 62% of mothers reported prenatal vitamin use during the first month of pregnancy, compared to 33% in ASD children (*p*-value < 0.001). A larger portion of TD children (65%) were born from mothers who owned a home, compared to ASD children (50%, p-value = 0.07). There was no difference between mother-child pairs included in the baseline MARBLES cohort (*n* = 286) and those included in the current study (*n* = 201) for any population characteristics (p-value > 0.05), providing evidence against selection bias due to follow-up related attrition (see Additional file 1: Table S4).

### Phthalate metabolite concentrations

The detection frequency for MHBP, MEHP, and MNP was 82, 83, and 50%, respectively, and the other 11 metabolites were detected in more than 90% of the samples (Table 2). The highest median was observed for MEP (22.0 μg/L), followed by MECPP (17.7 μg/L), MCOP (12.6 μg/L), and MBP (12.1 μg/L). Median concentrations for MiBP, MHiBP, MEHP, MEHHP, MEOHP, MECPP, MCPP, and MNP were significantly lower in the ASD group than in the TD group. Compared to TD children, maternal median concentrations for MBzP and ΣDEHP were significantly higher in Non-TD children. Median concentrations for FMVs and 24-h samples used as individual samples were not statistically different (see Additional file 1: Table S5). Geometric mean urinary concentrations of phthalate metabolites in the present study (pregnant mothers) were comparable, but slightly higher (see Additional file 1: Table S6) than those in the U.S. general female population (pregnant and non-pregnant) reported in the National Health and Nutrition Examination Survey (NHANES) [7].

**Table 2:** Distribution of SG-corrected phthalate metabolite concentrations (μg/L) in 636 urine samples collected from 198 pregnancies (201 mother-child pairs), the molar sum (μmol/L) of DEHP metabolites (ΣDEHP = MEHP + MEHHP + MEOHP + MECPP) and specific gravity (SG)

		LOD (μg/L)	% detect	All children (*n* = 208)			TD (*n* = 109)			ASD (*n* = 46)			Non-TD (*n* = 53)			ASD versus TD ^a^	Non-TD versus TD ^a^
				Percentiles			Percentiles			Percentiles			Percentiles				
				5	50	95	5	50	95	5	50	95	5	50	95		
DEP	MEP	1.2	100	5.5	22.0	193.5	5.5	21.7	173.3	6.1	20.1	181.8	5.5	25.7	195.8	0.61	0.25
DiBP	MiBP	0.8	98	1.9	7.1	23.3	2.3	7.4	24.2	1.7	6.0	19.1	1.9	7.3	23.2	0.00	0.94
	MHiBP	0.4	97	0.8	2.5	8.5	1.0	2.5	9.4	0.6	2.2	5.6	0.8	2.6	8.5	0.01	0.70
DnBP	MBP ^b^	0.4	99	3.4	12.1	40.9	3.8	12.3	37.4	2.4	11.2	48.1	3.5	12.4	44.0	0.21	0.73
	MHBP	0.4	82	<LOD	1.1	3.7	<LOD	1.1	3.5	<LOD	1.0	3.5	<LOD	1.1	4.3	0.25	0.34
BBP	MBzP	0.3	99	1.2	6.2	33.4	1.2	6.1	27.2	0.7	5.7	41.1	1.5	7.8	36.5	0.82	0.01
DEHP	MEHP	0.8	83	<LOD	2.5	25.7	<LOD	2.6	25.2	<LOD	2.2	18.0	<LOD	2.7	33.9	0.00	0.32
	MEHHP	0.4	100	2.5	11.3	105.9	3.2	11.7	108.1	1.4	9.6	70.4	2.4	11.8	119.3	0.00	0.44
	MEOHP	0.2	100	2.0	9.1	79.1	2.8	9.4	84.0	1.3	7.4	52.1	1.8	10.0	84.4	0.00	0.90
	MECPP	0.4	100	5.2	17.7	152.4	6.9	17.7	169.6	4.0	16.3	90.9	4.2	19.9	145.2	0.00	0.85
DnOP	MCPP ^c^	0.4	92	0.5	1.7	13.1	0.5	1.7	13.0	0.4	1.4	6.0	0.5	1.9	17.9	0.01	0.16
DNP	MNP	0.9	50	<LOD	1.1	8.1	<LOD	1.1	7.1	<LOD	0.9	5.2	<LOD	1.2	14.1	0.05	0.15
	MCOP	0.3	100	2.8	12.6	144.7	3.0	12.5	146.9	3.1	10.9	121.3	2.8	15.4	149.9	0.25	0.33
DDP	MCNP	0.2	100	0.8	2.6	22.9	0.8	2.6	15.4	0.7	2.3	18.0	0.9	2.9	34.5	0.38	0.12
ΣDEHP		–	–	0.05	0.17	1.48	0.06	0.17	1.51	0.03	0.14	0.90	0.04	0.19	1.53	0.00	0.04
Specific gravity		–	–	1.005	1.012	1.023	1.004	1.012	1.023	1.005	1.012	1.023	1.005	1.013	1.024	0.35	0.99

^a^P-value from the Wilcoxon rank-sum test of the null hypothesis that a randomly chosen value from members of one group is as likely to be greater than as to be less than a randomly chosen value from members of the comparison group. ^b^ MBP is also a minor metabolite of BBP. ^c^ MCPP is also a minor metabolite of DnBP and a non-specific metabolite of several high molecular weight phthalates

*Abbreviation*: *LOD* Limit of detection, *DEP* Diethyl phthalate, *DiBP* Di-isobutyl phthalate, *DnBP* Di-n-butyl phthalate, *BBP* Benzyl butyl phthalate, *DEHP* Di(2-ethylhexyl) phthalate, *DnOP* Di-n-octyl phthalate, *DNP* Di-isononyl phthalate, *DDP* Di-isodecyl phthalate, *MEP* Monoethyl phthalate, *MiBP* Mono-isobutyl phthalate, *MHiBP* Monohydroxy-isobutyl phthalate, *MBP* Mono-n-butyl phthalate, *MHBP* Monohydroxy-n-butyl phthalate, *MBzP* Monobenzyl phthalate, *MEHP* Mono(2-ethylhexyl) phthalate, *MEHHP* Mono(2-ethyl-5-hydroxyhexyl) phthalate, *MEOHP* Mono(2-ethyl-5-oxohexyl) phthalate, *MECPP* Mono(2-ethyl-5-carboxypentyl) phthalate, *MCPP* Mono(3-carboxypropyl) phthalate, *MNP* Mono-isononyl phthalate, *MCOP* Mono-carboxyisooctyl phthalate, *MCNP* Mono-carboxyisononyl phthalate

### Associations between prenatal phthalate exposures and the risk of ASD or non-TD

Overall, most associations of average concentrations of phthalate biomarkers during mid- to late pregnancy with both ASD and Non-TD were null. After adjusting for pre-pregnancy BMI, child’s birth year, and homeownership, none of the phthalate biomarkers showed statistically significant associations with ASD or Non-TD, with the exception that MEP was significantly associated with an increased risk of Non-TD (per 2.72-fold relative increase in concentration: Relative risk ratio (RRR) = 1.38; 95% confidence interval (CI): 1.01, 1.90, see top panel, Table 3). Two biomarkers (MCPP, MCNP) exhibited elevated RRRs for Non-TD risk with borderline significance (i.e., their lower confidence bounds were 0.95 and 0.96, respectively). When restricting the analyses to samples collected during the 2nd (*n* = 134) or 3rd trimesters (*n* = 190), the average phthalate biomarker concentrations from the 2nd trimester led to similar results, with the exception that MCPP was significantly associated with a decreased risk of ASD (RRR = 0.53; 95% CI: 0.32, 0.87, see middle panel, Table 3) and MCOP showed a reduced risk for ASD with borderline significance (RRR = 0.68; 95% CI: 0.46, 1.00). None of the phthalate biomarker concentrations from the 3rd trimester samples were associated with a risk of having a child with ASD or Non-TD (all *p*-values > 0.05, see bottom panel, Table 3). Additional adjustment for interpregnancy interval, gestational age at birth, maternal age, and child’s race/ethnicity led to similar results (see Additional file 1: Table S7). In addition, excluding top 2.5 percentiles of phthalate metabolite concentrations and 6 twins, and adjusting for the varying number of urine samples per mother did not change these results (see Additional file 1: Table S8).

**Table 3:** Relative risk ratios (RRR) from multinomial logistic regression and 95% confidence intervals (CI) of ASD and Non-TD (versus TD) in relation to phthalate metabolite concentrations (μg/L) during mid- to late pregnancy

	ASD versus TD		Non-TD versus TD	
	RRR ^a^	95% CI	RRR ^a^	95% CI
*Average exposure during the 2nd and 3rd trimesters* (*n* = 201; TD = 100, ASD = 46, Non-TD = 55)				
MEP	1.23	(0.81, 1.85)	1.38	(1.01, 1.90)*
MiBP	0.64	(0.37, 1.12)	1.18	(0.72, 1.94)
MHiBP	0.64	(0.35, 1.19)	1.10	(0.64, 1.90)
MBP	0.87	(0.45, 1.69)	1.48	(0.86, 2.53)
MHBP	0.91	(0.44, 1.89)	1.35	(0.77, 2.37)
MBzP	0.83	(0.53, 1.31)	1.31	(0.90, 1.90)
MCPP	0.71	(0.47, 1.06)	1.43	(0.95, 2.14)^#^
MCOP	0.76	(0.53, 1.11)	1.29	(0.89, 1.87)
MCNP	0.92	(0.61, 1.39)	1.38	(0.96, 1.97)^#^
ΣDEHP ^b^	0.75	(0.43, 1.32)	1.36	(0.88, 2.08)
*Analysis restricted to samples collected during the 2nd trimester* (*n* = 134; TD = 66, ASD = 29, Non-TD = 39)				
MEP	0.95	(0.61, 1.47)	1.34	(0.85, 2.10)
MiBP	0.53	(0.23, 1.19)	0.81	(0.40, 1.60)
MHiBP	0.56	(0.25, 1.21)	0.72	(0.33, 1.53)
MBP	0.48	(0.19, 1.16)	0.81	(0.42, 1.56)
MHBP	0.54	(0.24, 1.21)	0.82	(0.42, 1.59)
MBzP	0.75	(0.42, 1.32)	1.31	(0.79, 2.15)
MCPP	0.53	(0.32, 0.87)*	1.31	(0.83, 2.07)
MCOP	0.68	(0.46, 1.00)	1.21	(0.77, 1.87)
MCNP	0.79	(0.49, 1.25)	1.24	(0.80, 1.91)
ΣDEHP ^b^	0.81	(0.45, 1.42)	1.14	(0.71, 1.82)
*Analysis restricted to samples collected during the 3rd trimester* (*n* = 190; TD = 97, ASD = 43, Non-TD = 50)				
MEP	1.30	(0.84, 1.98)	1.27	(0.92, 1.72)
MiBP	0.60	(0.33, 1.08)	1.21	(0.73, 1.98)
MHiBP	0.60	(0.32, 1.10)	1.10	(0.63, 1.92)
MBP	1.11	(0.55, 2.21)	1.54	(0.93, 2.55)
MHBP	1.32	(0.67, 2.59)	1.52	(0.88, 2.60)
MBzP	0.94	(0.61, 1.43)	1.33	(0.92, 1.92)
MCPP	0.86	(0.58, 1.28)	1.17	(0.80, 1.69)
MCOP	0.85	(0.59, 1.20)	1.01	(0.71, 1.42)
MCNP	0.90	(0.59, 1.35)	1.19	(0.82, 1.70)
ΣDEHP ^b^	0.83	(0.44, 1.54)	1.28	(0.83, 1.94)

^a^Adjusted for pre-pregnancy BMI, year of birth (linear and squared terms), and homeownership

For a binary outcome, odds ratios would correspond to relative risk ratios (RRRs). In either case, ratios of relative risks are being described, with the relative risk being equal to the probability of having the stated level of the outcome divided by the probability of having the reference level of the outcome. The metabolites were measured in μg/L and were then natural log-transformed. Hence, the RRR corresponds to how much the relative risk changes (relatively) for a 2.72-fold increase in the metabolite concentration

^b^Because correlations among DEHP metabolites were high (*r* = 0.84–0.99), the molar sum of DEHP metabolites was only examined (ΣDEHP, μmol/L)

*p-value < 0.05, # *p*-value < 0.10

Note: MNP was excluded from this table because approximately 50% of samples had detectable concentrations. See Table 2 for abbreviations

Results from models stratified by prenatal vitamin use during the first month of pregnancy showed that mothers who took prenatal vitamins (*n* = 105) had a reduced risk of delivering a child that developed ASD in association with MiBP (RRR = 0.44; 95% CI: 0.21, 0.88), MCPP (RRR = 0.41; 95% CI: 0.20, 0.83), and MCOP (RRR = 0.49; 95% CI: 0.27, 0.88) (Table 4). In contrast, among mothers who did *not* take prenatal vitamins during the first month of pregnancy (*n* = 85), RRRs for ASD risk did not reach statistical significance for all biomarker concentrations. On the other hand, risk of Non-TD was increased in association with MCPP (RRR = 5.09; 95% CI: 2.05, 12.6), MCOP (RRR = 1.86; 95% CI: 1.01, 3.39), and MCNP (RRR = 3.67; 95% CI: 1.80, 7.48) for children of mothers who did *not* take prenatal vitamins during the first month of pregnancy.

**Table 4:** Relative risk ratios (RRR) from multinomial logistic regression and 95% confidence intervals (CI) of ASD and Non-TD (versus TD) in relation to phthalate metabolite concentrations (μg/L) during mid- to late pregnancy among mothers who took prenatal vitamins during the first month of pregnancy and those who did not

	Prenatal vitamin use (*n* = 105; TD = 61, ASD = 15, Non-TD = 29)		No prenatal vitamin use (*n* = 85; TD = 36, ASD = 26, Non-TD = 23)		*P*-value for interaction ^c^
	RRR ^a^	95% CI	RRR ^a^	95% CI	
*ASD* versus *TD*					
MEP	1.88	(0.82, 4.27)	0.77	(0.42, 1.37)	0.12
MiBP	0.44	(0.21, 0.88)*	0.71	(0.32, 1.51)	0.52
MHiBP	0.52	(0.22, 1.20)	0.61	(0.27, 1.35)	0.97
MBP	0.48	(0.17, 1.30)	0.90	(0.35, 2.26)	0.33
MHBP	0.53	(0.18, 1.56)	0.87	(0.31, 2.36)	0.43
MBzP	0.85	(0.41, 1.73)	0.69	(0.37, 1.30)	0.97
MCPP	0.41	(0.20, 0.83)*	1.33	(0.60, 2.93)	0.02
MCOP	0.49	(0.27, 0.88)*	1.17	(0.66, 2.05)	0.08
MCNP	0.85	(0.50, 1.42)	1.16	(0.55, 2.43)	0.47
ΣDEHP ^b^	0.51	(0.19, 1.36)	1.14	(0.50, 2.56)	0.12
*Non-TD* versus *TD*					
MEP	1.53	(0.97, 2.41)^#^	1.28	(0.76, 2.14)	0.79
MiBP	1.18	(0.51, 2.70)	1.39	(0.68, 2.83)	0.84
MHiBP	1.20	(0.45, 3.16)	1.24	(0.61, 2.50)	1.00
MBP	1.48	(0.63, 3.46)	1.86	(0.75, 4.61)	0.66
MHBP	1.38	(0.57, 3.32)	1.87	(0.70, 4.97)	0.72
MBzP	1.47	(0.82, 2.62)	1.16	(0.65, 2.07)	0.74
MCPP	0.93	(0.54, 1.57)	5.09	(2.05, 12.6)*	0.00
MCOP	0.99	(0.58, 1.66)	1.86	(1.01, 3.39)*	0.11
MCNP	0.88	(0.53, 1.46)	3.67	(1.80, 7.48)*	0.00
ΣDEHP ^b^	1.26	(0.72, 2.19)	1.76	(0.84, 3.65)	0.30

^a^Adjusted for pre-pregnancy BMI, year of birth (linear and squared terms), and homeownership

^b^Due to high correlations among DEHP metabolites (*r* = 0.84–0.99), the molar sum of DEHP metabolites was only examined (ΣDEHP, μmol/L)

^c^*P*-value for interaction from the separate regression models

*p-value < 0.05, # *p*-value < 0.10

Note: MNP was excluded from this table because approximately 50% of samples had detectable concentrations. See Table 2 for abbreviations

When stratified by sex, among boys (*n* = 122), RRRs for ASD risk did not reach statistical significance for all biomarker concentrations. However, MEP, MBzP, MCPP, MCNP, and ΣDEHP were positively associated with Non-TD risk (see Table 5 for RRRs and *p*-values). Among girls (*n* = 79), associations of prenatal phthalate biomarkers with both ASD and Non-TD were null.

**Table 5:** Relative risk ratios (RRR) and 95% confidence intervals (CI) of ASD and Non-TD (versus TD) in relation to phthalate metabolite concentrations (μg/L) during mid- to late pregnancy among boys and girls

	Girls (*n* = 79; TD = 46, ASD = 13, Non-TD = 20)		Boys (*n* = 122; TD = 54, ASD = 33, Non-TD = 35)		P-value for interaction ^c^
	RRR ^a^	95% CI	RRR ^a^	95% CI	
*ASD* versus *TD*					
MEP	0.58	(0.26, 1.29)	1.46	(0.89, 2.37)	0.01
MiBP	0.75	(0.22, 2.48)	0.48	(0.22, 1.02)	0.33
MHiBP	0.71	(0.20, 2.52)	0.52	(0.24, 1.10)	0.48
MBP	1.08	(0.27, 4.16)	0.82	(0.37, 1.80)	0.79
MHBP	0.84	(0.18, 3.80)	0.96	(0.41, 2.19)	0.97
MBzP	0.59	(0.27, 1.27)	1.01	(0.57, 1.77)	0.27
MCPP	0.74	(0.38, 1.42)	0.86	(0.47, 1.57)	0.67
MCOP	0.82	(0.47, 1.41)	0.84	(0.50, 1.38)	0.78
MCNP	0.76	(0.41, 1.39)	1.28	(0.63, 2.58)	0.17
ΣDEHP ^b^	1.18	(0.28, 5.01)	0.85	(0.43, 1.63)	0.36
*Non-TD* versus *TD*					
MEP	1.08	(0.57, 2.02)	1.54	(1.01, 2.35)*	0.25
MiBP	1.43	(0.68, 2.96)	1.02	(0.52, 2.00)	0.49
MHiBP	1.81	(0.74, 4.39)	0.90	(0.45, 1.78)	0.30
MBP	1.39	(0.59, 3.23)	1.59	(0.76, 3.33)	0.92
MHBP	1.20	(0.41, 3.47)	1.54	(0.77, 3.07)	0.62
MBzP	0.86	(0.47, 1.58)	1.75	(1.06, 2.88)*	0.15
MCPP	1.26	(0.63, 2.49)	1.99	(1.04, 3.77)*	0.18
MCOP	1.28	(0.69, 2.35)	1.37	(0.81, 2.30)	0.86
MCNP	1.10	(0.67, 1.78)	1.85	(1.09, 3.13)*	0.15
ΣDEHP ^b^	1.16	(0.54, 2.46)	1.87	(1.02, 3.41)*	0.13

^a^Adjusted for pre-pregnancy BMI, year of birth (linear and squared terms), and homeownership

^b^Due to high correlations among DEHP metabolites (*r* = 0.84–0.99), the molar sum of DEHP metabolites was only examined (ΣDEHP, μmol/L)

^c^*P*-value for interaction from the separate regression models

*p-value < 0.05

Note: MNP was excluded from this table because approximately 50% of samples had detectable concentrations. See Table 2 for abbreviations

When our analyses were further stratified by prenatal vitamin use and by child sex with the trimester-specific phthalate biomarker concentrations (i.e., average concentrations during the 2nd or 3rd trimesters), among mothers who took prenatal vitamins, we observed that exposure to MiBP, MHiBP, MCPP, and MCOP during the 2nd trimester was significantly associated with a decreased risk of ASD, and exposure to MEP during the 3rd trimester was significantly associated with an increased risk of Non-TD (see Additional file 1: Tables S9-S10 for RRRs and 95% CIs). Among mothers who did *not* take prenatal vitamins, exposure to MCNP during the 2nd trimester and exposure to MCPP and MCNP during the 3rd trimester were significantly associated with an increased risk of Non-TD and associations of prenatal phthalate biomarkers with ASD were null. Among boys, exposure to MiBP, MHiBP, and MCPP during the 2nd trimester was significantly associated with a decreased risk of ASD, and exposure to MEP during the 2nd trimester and exposure to MBzP during the 3rd trimester were significantly associated with an increased risk of Non-TD (see Additional file 1: Tables S11-S12 for RRRs and 95% CIs). Among girls, associations of trimester-specific concentrations of prenatal phthalate biomarkers with both ASD and Non-TD were null.

## Discussion

Results from this study did not support our hypothesis that phthalate exposures during mid- to late pregnancy are associated with an increased risk of having a child with ASD in overall analyses, but showed that exposures to some phthalates may increase risk of having a child with Non-TD. MEP was statistically significant, and MCPP and MCNP showed borderline significance. Except for MEP, our null findings for ASD and Non-TD risks might be explained by the moderately small number of ASD (*n* = 46) and Non-TD (*n* = 53), because heterogeneity in the associations with statistical significance was found for some biomarkers in stratified analyses. When analyses were stratified by child’s sex, we observed opposite directions of RRRs of ASD between girls and boys for four metabolites (i.e., MEP, MBP, MBzP, MCNP) and ΣDEHP. This is additional evidence for sex dimorphism of childhood neurodevelopment associated with prenatal phthalate exposures [58, 63–66].

Heterogeneity in the associations was also found when analyses were stratified by prenatal multivitamin supplement use during the first month of pregnancy. Supplemental folic acid taken near conception has previously been shown to modify associations between environmental contaminants and ASD [53, 54]. We observed that prenatal phthalate biomarkers tended to be associated with a decreased risk of ASD among mothers who took prenatal vitamins with statistical significance at the 5% level reached for three metabolites (i.e., MiBP, MCPP, MCOP), whereas associations for these compounds were null among mothers who did *not* take prenatal vitamins. In addition, we observed that exposures to MCPP, MCOP, and MCNP were associated with an increased risk of Non-TD only among mothers who did *not* take prenatal vitamins.

Though these findings for heterogeneity should be interpreted with caution given the limited number of ASD and Non-TD children and multiple comparisons, there is biologic plausibility for such interaction effects. Potential biologic mechanisms for effect modification by prenatal multivitamin intake could include compensation for direct effects of phthalates on fetal micronutrient availability, because DEHP and other phthalates have been linked with deficiencies of vitamin D [67] and zinc [68–70], that are often included in prenatal vitamins. Steroid metabolism and growth effects of gestational phthalate exposure are amplified when paired with zinc deficiency in rats [56]. DEHP may also disrupt fetal micronutrient homeostasis by stimulating a maternal inflammatory response [71, 72]. Several nutrients can become depleted in inflammatory states [73], and vitamins B, C, D and E, that are usually contained in prenatal vitamins, have anti-inflammatory properties [74], including in the brain [75–77]. Anti-inflammatory and anti-oxidant vitamins like vitamin E [78] have been shown to protect against the neurobehavioral effects of DEHP in mice [59]. The biologic effects of phthalates and certain nutrients found in prenatal multivitamins could also converge on shared physiologic pathways, like hormone or epigenetic pathways, but in opposite directions.

Environmental phthalate exposures could potentially alter circulating levels of thyroid hormones in pregnant women [27], and several minerals and trace elements typically contained in prenatal multivitamins are essential for normal thyroid hormone metabolism, including iodine, iron, selenium, and zinc [79]. Prenatal phthalate exposures have also been linked to altered DNA methylation patterns in both human placenta [80] and cord blood [81], and the effects of phthalate exposures on asthma are thought to be mediated through DNA methylation alterations [82]. Similarly, folic acid and other B-vitamins that are contained at high concentrations in prenatal vitamins are essential for DNA methylation reactions [83, 84], and prenatal folate is associated with altered placental [85] and cord DNA methylation [86]. Examining effect modification by specific nutrients could shed light on potential mechanisms involved. Further studies using phthalate exposures characterized from the first trimester samples, closer to the time of epigenetic reprogramming, are also warranted.

To our knowledge no previous research has evaluated the association between prenatal exposure to phthalates and an ASD diagnosis. Because phthalate metabolites measured in humans are only indicative of recent exposure, having multiple gestational biological samples is critical for robust phthalate exposure assessment. The MARBLES study can provide useful data for an investigation of phthalates in relation to risk for ASD because it has followed pregnant women and prospectively collected their biological samples at multiple time points over pregnancy. However, because the MARBLES participants were at elevated risk for ASD because of their family history of this condition, results from this study may not extrapolate to the general population who are not at high risk for ASD. The MARBLES children are, on average, likely to have a much higher genetic susceptibility than the general population, and therefore results from this study may not represent the general population susceptibility for ASD in relation to exposure to phthalates. It may be more difficult to find relationships between phthalate exposures and ASD in this population due to the strong genetic components.

Strengths of this study include (1) clinically confirmed diagnostic classification (ASD, Non-TD, TD) based on gold standard diagnostic assessments and (2) adjustment to confounding factors prospectively collected during pregnancy and obtained from literature review. Sensitivity analyses by excluding extreme values and twins as well as additional adjustment for other variables did not change our results, demonstrating robustness of the results as further evidence of the study reliability. Another strength of this study is the strong exposure assessment strategy based on multiple biomarker measurements in the 2nd and 3rd trimesters. More than two thirds of mothers who participated in this study provided four or more samples during mid- to late pregnancy (5 samples were collected per mother on average). Multiple FMVs help increase reproducibility of an individual’s exposure, compared to a single spot sample, because phthalates are quickly metabolized and excreted in urine. From the variability analysis with two samples of the same urine type (i.e., two FMVs or two pools) collected across the 2nd and 3rd trimesters, we confirmed that within-subject pooling resulted in more reproducible individual’s exposure (as measured by intraclass correlation coefficient (ICCs) than FMVs (see Additional file 1: Figure S5) [87]. This indicates that within-subject pools can improve phthalate exposure characterization. In spite of this improved exposure assessment strategy, the average exposure characterized from multiple (4 or 5) samples might not necessarily represent the individual’s true exposure for compounds with low ICCs (e.g., MEHP, MEHHP, MEOHP, MECPP, MCPP, MNP, MCOP, MCNP), whose molecular weight is relatively high and for which diet is a primary exposure source. For example, for phthalate metabolites with low ICCs, Spearman’s correlation coefficients between the average concentrations of the 2nd and those of the 3rd trimesters were only between 0.07 (MCPP) and 0.46 (MEOHP) (see Additional file 1: Table S13), indicating high within-subject variability during mid- to late pregnancy. This implies that our results should be interpreted with caution for biomarkers with low ICCs.

## Conclusions

In this study of children at high risk for neurodevelopmental deficits, analysis of prenatal phthalate exposures produced varying findings including both reduced and elevated risks, which may be due to random error as a result of the small number of cases. Because these children had family members with ASD, results may not be generalizable to the broader population. The null and unexpected inverse associations may have been related to substantial exposure misclassification from the episodic nature of exposures to phthalates or to a potentially strong genetic component to ASD in this high-risk population. Further studies should be conducted in the general population without high-risk genes to confirm our findings. We also observed sex-specific associations and heterogeneous results based on maternal prenatal vitamin intake. Our results are additional evidence for sex dimorphism of childhood neurodevelopment associated with prenatal phthalate exposures [58, 63–66]. We additionally found hypothesized positive associations between prenatal concentrations of three phthalate metabolites (MCPP, MCOP, MCNP) and an increased risk of Non-TD among mothers who did *not* take prenatal vitamins, whereas inverse associations were found between prenatal concentrations of three phthalate metabolites (MiBP, MCPP, MCOP) and an increased risk of ASD among mothers taking prenatal vitamins. These findings add to previous reports supporting beneficial effects of prenatal vitamins in early pregnancy, especially for mothers exposed to environmental chemicals [53, 54, 88]. Future work is needed to confirm these findings and to elucidate mechanisms for such interactions between phthalates and prenatal vitamin in relation to the risk of ASD. Although urinary concentrations of 14 phthalate metabolites were measured, the regression models used in this study were applied to individual metabolites or the molar sum of DEHP, neglecting potential co-pollutant confounding by other phthalate metabolites. Thus, further studies are needed to assess influence of the confounding by other phthalate metabolites on our findings using a multipollutant model [89, 90].

## Additional file

Additional file 1: **Table S1.** Summary of all possible combinations of individual and pooled samples analyzed per mother. **Table S2.** Distribution of specific gravity for each type of urine samples. **Table S3.** Pearson’s correlations for ln-transformed phthalate metabolite concentrations. **Table S4.** Characteristics of study population included in this study and MARBLES. **Table S5.** Comparison between FMVs and 24-hour samples used as individual samples. **Table S6.** Comparison of phthalate metabolite concentrations in the current study and those in NHANES. **Table S7.** Sensitivity analyses I: RRR for ASD and Non-TD (versus TD) in relation to phthalate biomarker concentrations during mid- to late pregnancy. **Table S8.** Sensitivity analyses II: RRR for ASD and Non-TD (versus TD) in relation to phthalate biomarker concentrations during mid- to late pregnancy. **Table S9.** Stratified analyses by prenatal vitamin use during the first month of pregnancy using samples during mid-pregnancy. **Table S10.** Stratified analyses by prenatal vitamin use during the first month of pregnancy using samples during late pregnancy a. **Table S11.** Stratified analyses by sex using samples during mid-pregnancys. **Table S12.** Stratified analyses by sex using samples during late pregnancy. **Table S13.** Spearman’s correlations average phthalate metabolite concentrations between the 2^nd^ trimester samples (*n* =123) and the 3^rd^ trimester samples (*n* =123). **Figure S1.** Selection of mother-child pairs used in this study. **Figure S2.** Type and number of urine samples analyzed in this study. **Figure S3.** Directed acyclic graph used to identify and select adjustment factors. **Figure S4.** Temporal trends of median phthalate biomarker concentrations (in μg/L) in the selected MARBLES participants. **Figure S5.** ICC and 95% CI of ln-transformed phthalate metabolite concentrations using samples collected during each of the 2^nd^ and 3^rd^ trimesters: (1) two first FMVs (*n* = 204 from 102 mothers) and (2) two pooled samples (*n* = 118 from 59 mothers). (DOCX 516 kb)

## Abbreviations

ADHD: Attention deficit hyperactivity disorder
ADOS: Autism Diagnostic Observation Schedules
ASD: Autism spectrum disorder
BMI: Body mass index
CDC: Centers for Disease Control and Prevention
CI: Confidence interval
DAG: Directed acyclic graph
DBP: Dibutyl phthalate
DEHP: Di(2-ethylhexyl) phthalate
DEP: Diethyl phthalate
DSM: Diagnostic and Statistical Manual of Mental Disorders
EDC: Endocrine disrupting chemicals
FMV: First morning voids
ICC: Intraclass correlation coefficient
LOD: Limit of detection
MARBLES: Markers of Autism Risk in Babies – Learning Early Signs
MBP: Mono-n-butyl phthalate
MBzP: Monobenzyl phthalate
MCNP: Mono-carboxyisononyl phthalate
MCOP: Mono-carboxyisooctyl phthalate
MCPP: Mono(3-carboxypropyl) phthalate
MECPP: Mono(2-ethyl-5-carboxypentyl) phthalate
MEHHP: Mono(2-ethyl-5-hydroxyhexyl) phthalate
MEHP: Mono(2-ethylhexyl) phthalate
MEOHP: Mono(2-ethyl-5-oxohexyl) phthalate
MEP: Monoethyl phthalate
MHBP: Monohydroxy-n-butyl phthalate
MHiBP: Monohydroxy-isobutyl phthalate
MiBP: Mono-isobutyl phthalate
MNP: Mono-isononyl phthalate
MSEL: Mullen Scales of Early Learning
NHANES: National Health and Nutrition Examination Survey
Non-TD: Non-typical development
PVC: Polyvinyl chloride
RRR: Relative risk ratio
SG: Specific gravity
TD: Typical development
