# Supplementary material for: Prenatal exposure to phthalates and autism spectrum disorder in the MARBLES study
Source: Environ Health. 2018 Dec 5;17:85. doi: 10.1186/s12940-018-0428-4 (PMC6280477; doi:10.1186/s12940-018-0428-4)
Supplement: Supplementary file 1 — Table S1. Summary of all possible combinations of individual and pooled samples analyzed per mother. Table S2. Distribution of specific gravity for each type of urine samples. Table S3. Pearson’s correlations for ln-transformed phthalate metabolite concentrations. Table S4. Characteristics of study population included in this study and MARBLES. Table S5. Comparison between FMVs and 24-hour samples used as individual samples. Table S6. Comparison of phthalate metabolite concentrations in the current study and those in NHANES. Table S7. Sensitivity analyses I: RRR for ASD and Non-TD (versus TD) in relation to phthalate biomarker concentrations during mid- to late pregnancy. Table S8. Sensitivity analyses II: RRR for ASD and Non-TD (versus TD) in relation to phthalate biomarker concentrations during mid- to late pregnancy. Table S9. Stratified analyses by prenatal vitamin use during the first month of pregnancy using samples during mid-pregnancy. Table S10. Stratified analyses by prenatal vitamin use during the first month of pregnancy using samples during late pregnancy a. Table S11. Stratified analyses by sex using samples during mid-pregnancys. Table S12. Stratified analyses by sex using samples during late pregnancy. Table S13. Spearman’s correlations average phthalate metabolite concentrations between the 2nd trimester samples (n =123) and the 3rd trimester samples (n =123). Figure S1. Selection of mother-child pairs used in this study. Figure S2. Type and number of urine samples analyzed in this study. Figure S3. Directed acyclic graph used to identify and select adjustment factors. Figure S4. Temporal trends of median phthalate biomarker concentrations (in μg/L) in the selected MARBLES participants. Figure S5. ICC and 95% CI of ln-transformed phthalate metabolite concentrations using samples collected during each of the 2nd and 3rd trimesters: (1) two first FMVs (n = 204 from 102 mothers) and (2) two pooled samples (n = 118 from 59 mothers). (DOCX 516 kb) [file 12940_2018_428_MOESM1_ESM.docx]

Additional File

**Prenatal Exposure to Phthalates and Autism Spectrum Disorders in the MARBLES Study**

Hyeong-Moo Shin ^a, b*^, Rebecca Schmidt ^a^, Daniel Tancredi ^c^, Jacqueline Barkoski ^a^, Sally Ozonoff ^d, e^, Deborah H. Bennett ^a^, Irva Hertz-Picciotto ^a, d^

^a^ Department of Public Health Sciences, University of California, Davis, California, USA

^b^ Department of Earth and Environmental Sciences, University of Texas, Arlington, Texas, USA

^c^ Department of Pediatrics, University of California, Davis, California, USA

^d^ UC Davis MIND (Medical Investigations of Neurodevelopmental Disorders) Institute, Sacramento, California, USA

^e^ Department of Psychiatry and Behavioral Sciences, University of California Davis Medical Center, Sacramento, California, USA

*Corresponding author (present address):

Hyeong-Moo Shin, Ph.D.

University of Texas, Arlington

500 Yates Street, Box 19049

Arlington, Texas 76019

Email: [hyeongmoo.shin@uta.edu](mailto:hmshin@ucdavis.edu)

Voice: 817-272-2970

Fax: 817-272-2628

**Table S1**. All possible combinations of individual and pooled samples analyzed per mother.

| Case | Number of samples mothers provided | Number of analyzed samples per mother | Number of mothers |
| --- | --- | --- | --- |
| 1 | One sample during entire pregnancy | **1** individual sample | 17 |
| 2 | Two samples during one of 2^nd^ or 3^rd^ trimesters | **2** individual samples | 16 |
| 3 | One sample for each of 2^nd^ and 3^rd^ trimesters | **2** individual samples | 6 |
| 4 | Two samples during one of two trimesters  and one sample during the other trimester | **3** individual samples | 5 |
| 5 | Two samples for both 2^nd^ and 3^rd^ trimesters | **4** individual samples | 3 |
| 6 | More than two samples during one of two  trimesters only | **1** individual samples  **1** pooled sample | 33 |
| 7 | More than two samples during one of two  trimesters and one sample during the other trimester | **2** individual samples  **1** pooled sample | 29 |
| 8 | More than two samples during one of two  trimesters and two samples during the other trimester | **3** individual samples  **1** pooled sample | 23 |
| 9 | More than two samples for both trimesters | **2** individual samples  **2** pooled samples | 55 |
| 10 | At least four samples for both trimesters | **8** or **9** individual samples | 9 ^*^ |
| 11 | More than two samples for both trimesters, but the first individual was later determined to be 1^st^ trimester | **1** individual samples  **2** pooled sample | 1 |
| 12 | More than two samples for both trimesters and two individual samples were sent from one trimester | **3** individual samples  **2** pooled sample | 1 |

**Note:** In some cases, samples provided by mothers as the 2^nd^ trimester sample were later determined as the 3^rd^ trimester sample or vice versa. In other cases, mothers just collected samples more than necessary.

*For these nine mothers, we did not pool their samples, but analyzed them individually to evaluate the concentration variability over the course of the 2^nd^ and 3^rd^ trimesters. The results of this variability analysis are available elsewhere (Shin et al. under review).

**Table S2**. Distribution of specific gravity for each type of urine samples.

|  | All samples | First morning voids | 24-hour | Pooled samples |
| --- | --- | --- | --- | --- |
| N | 636 | 355 | 82 | 199 |
| minimum | 1.001 | 1.001 | 1.004 | 1.002 |
| 5th percentile | 1.005 | 1.004 | 1.005 | 1.006 |
| 25th percentile | 1.009 | 1.008 | 1.008 | 1.009 |
| 50th percentile | 1.012 | 1.012 | 1.011 | 1.012 |
| 75th percentile | 1.017 | 1.017 | 1.016 | 1.016 |
| 95th percentile | 1.023 | 1.025 | 1.022 | 1.021 |
| maximum | 1.033 | 1.033 | 1.029 | 1.027 |
| mean | 1.013 | 1.013 | 1.012 | 1.013 |
| geometric mean | 1.013 | 1.013 | 1.012 | 1.013 |
| standard deviation | 0.006 | 0.006 | 0.005 | 0.005 |
| coefficient of variation | 0.006 | 0.006 | 0.005 | 0.005 |
| ICC |  | 0.433 | 0.505 | 0.703 |
| ICC 95% CI |  | (0.30, 0.55) | (0.01, 0.81) | (0.55, 0.81) |

**Note:** For intracclass correlation (ICC) calculation, we only included mothers who provided at least two samples of the same type per pregnancy. The sample size for ICC calculations for first morning voids, 24-hour samples, and pooled samples in this table is 293, 28, and 118, respectively.

**Table S3.** Pearson’s correlations for ln-transformed phthalate metabolite concentrations.

|  | MEP | MiBP | MHiBP | MBP | MHBP | MBzP | MEHP | MEHHP | MEOHP | MECPP | MCPP | MNP | MCOP | MCNP |
| --- | --- | --- | --- | --- | --- | --- | --- | --- | --- | --- | --- | --- | --- | --- |
| MEP | 1 |  |  |  |  |  |  |  |  |  |  |  |  |  |
| MiBP | 0.16 | 1 |  |  |  |  |  |  |  |  |  |  |  |  |
| MHiBP | 0.14 | **0.90** | 1 |  |  |  |  |  |  |  |  |  |  |  |
| MBP | 0.25 | 0.61 | 0.53 | 1 |  |  |  |  |  |  |  |  |  |  |
| MHBP | 0.23 | 0.51 | 0.61 | **0.84** | 1 |  |  |  |  |  |  |  |  |  |
| MBzP | 0.14 | 0.28 | 0.27 | 0.60 | 0.55 | 1 |  |  |  |  |  |  |  |  |
| MEHP | 0.20 | 0.13 | 0.06 | 0.23 | 0.13 | 0.12 | 1 |  |  |  |  |  |  |  |
| MEHHP | 0.22 | 0.21 | 0.17 | 0.30 | 0.22 | 0.20 | **0.88** | 1 |  |  |  |  |  |  |
| MEOHP | 0.23 | 0.23 | 0.20 | 0.32 | 0.26 | 0.22 | **0.87** | **0.99** | 1 |  |  |  |  |  |
| MECPP | 0.22 | 0.18 | 0.13 | 0.26 | 0.16 | 0.12 | **0.84** | **0.96** | **0.96** | 1 |  |  |  |  |
| MCPP | 0.00 | 0.27 | 0.28 | 0.25 | 0.25 | 0.15 | 0.14 | 0.19 | 0.19 | 0.24 | 1 |  |  |  |
| MNP | 0.00 | 0.18 | 0.12 | 0.14 | 0.09 | 0.03 | 0.29 | 0.19 | 0.18 | 0.20 | 0.65 | 1 |  |  |
| MCOP | 0.01 | 0.23 | 0.19 | 0.14 | 0.10 | 0.03 | 0.10 | 0.14 | 0.14 | 0.19 | **0.77** | **0.79** | 1 |  |
| MCNP | 0.08 | 0.13 | 0.11 | 0.04 | 0.00 | -0.04 | 0.09 | 0.12 | 0.13 | 0.24 | 0.55 | 0.34 | 0.48 | 1 |

**Table S4**. Characteristics of study population included in this study and MARBLES.

|  | Row total | **Current study** ^a^ | **MARBLES** | **Current study** | **MARBLES** |  | Current  versus  MARBLES ^b^ |
| --- | --- | --- | --- | --- | --- | --- | --- |
| **Characteristics** | *n* | *n* | | % | |  |  |
| **Child's diagnosis** |  |  |  |  |  |  | 0.85 |
| TD | 206 | 100 | 106 | 50% | 37% |  |  |
| ASD | 94 | 46 | 48 | 23% | 17% |  |  |
| Non-TD | 110 | 55 | 55 | 27% | 19% |  |  |
| Missing | 77 | 0 | 77 | 0% | 27% |  |  |
| **Child's sex** |  |  |  |  |  |  | 0.43 |
| Girl | 189 | 79 | 110 | 39% | 38% |  |  |
| Boy | 268 | 122 | 146 | 61% | 51% |  |  |
| Missing | 30 | 0 | 30 | 0% | 10% |  |  |
| **Birth year** |  |  |  |  |  |  | 0.76 |
| 2007 | 34 | 11 | 23 | 6% | 8% |  |  |
| 2008 | 76 | 35 | 41 | 18% | 14% |  |  |
| 2009 | 104 | 46 | 58 | 23% | 20% |  |  |
| 2010 | 77 | 31 | 46 | 16% | 16% |  |  |
| 2011 | 45 | 19 | 26 | 10% | 9% |  |  |
| 2012 | 38 | 17 | 21 | 9% | 7% |  |  |
| 2013 | 70 | 34 | 36 | 17% | 13% |  |  |
| 2014 | 8 | 5 | 3 | 3% | 1% |  |  |
| **Child's race/ethnicity** | |  |  |  |  |  | 0.98 |
| White (non-Hispanic) | 188 | 89 | 99 | 44% | 35% |  |  |
| Hispanic | 124 | 60 | 64 | 30% | 22% |  |  |
| Other ^c^ | 109 | 52 | 57 | 26% | 20% |  |  |
| Missing | 66 | 0 | 66 | 0% | 23% |  |  |
| **Birth Status** | |  |  |  |  |  | 0.67 |
| Singleton | 445 | 195 | 250 | 97% | 87% |  |  |
| Has a twin | 12 | 6 | 6 | 3% | 2% |  |  |
| Missing | 30 | 0 | 30 | 0% | 10% |  |  |
| **Gestational age at delivery** | |  |  |  |  |  | 0.91 |
| ≤ 37 weeks | 61 | 18 | 43 | 9% | 15% |  |  |
| > 37 weeks | 422 | 180 | 242 | 91% | 85% |  |  |
| Missing | 1 | 0 | 1 | 0% | 0% |  |  |
| **Maternal pre-pregnancy BMI** | |  |  |  |  |  | 0.93 |
| Normal/ underweight | 212 | 103 | 109 | 52% | 38% |  |  |
| Overweight | 102 | 48 | 54 | 24% | 19% |  |  |
| Obese | 103 | 46 | 57 | 23% | 20% |  |  |
| Missing | 67 | 1 | 66 | 1% | 23% |  |  |
| **Maximum parental education** | |  |  |  |  |  | 0.56 |
| Less than college degree | 136 | 62 | 74 | 32% | 26% |  |  |
| Bachelor’s degree | 176 | 87 | 89 | 45% | 31% |  |  |
| Graduate or professional degree | 100 | 43 | 57 | 22% | 20% |  |  |
| Missing | 66 | 0 | 66 | 0% | 23% |  |  |
| **Mother's age at delivery** | |  |  |  |  |  | 0.76 |
| < 35 years | 225 | 105 | 120 | 53% | 42% |  |  |
| ≥ 35 years | 193 | 93 | 100 | 47% | 35% |  |  |
| Missing | 66 | 0 | 66 | 0% | 23% |  |  |
| **Father's age at delivery** |  |  |  |  |  |  | 0.96 |
| < 35 years | 162 | 75 | 87 | 38% | 30% |  |  |
| ≥ 35 years | 247 | 118 | 129 | 60% | 45% |  |  |
| Missing | 75 | 5 | 70 | 3% | 24% |  |  |
| **Prenatal vitamin use in the first month of pregnancy** | |  |  |  |  |  | 0.25 |
| Yes | 223 | 105 | 118 | 53% | 41% |  |  |
| No | 217 | 85 | 132 | 43% | 46% |  |  |
| Missing | 44 | 8 | 36 | 4% | 13% |  |  |
| **Homeowner** |  |  |  |  |  |  | 0.96 |
| Yes | 245 | 117 | 128 | 59% | 45% |  |  |
| No | 162 | 75 | 87 | 38% | 30% |  |  |
| Missing | 77 | 6 | 71 | 3% | 25% |  |  |
| **Parity** |  |  |  |  |  |  | 0.98 |
| 0 ^d^ | 4 | 2 | 2 | 1% | 1% |  |  |
| 1 | 181 | 80 | 101 | 40% | 35% |  |  |
| >1 | 257 | 110 | 147 | 56% | 51% |  |  |
| Missing | 42 | 6 | 36 | 3% | 13% |  |  |
| **Mom ever smoked** | |  |  |  |  |  | 0.66 |
| Yes | 165 | 70 | 95 | 35% | 33% |  |  |
| No | 276 | 123 | 153 | 62% | 53% |  |  |
| Missing | 43 | 5 | 38 | 3% | 13% |  |  |
| **Season of conception** (months) | |  |  |  |  |  | 0.43 |
| Cold (November – April) | 100 | 100 | 134 | 51% | 47% |  |  |
| Warm (May – October) | 98 | 98 | 152 | 49% | 53% |  |  |
| **Number of urine samples collected** | |  |  |  |  |  | 0.96 |
| 1 – 2 | 57 | 26 | 31 | 13% | 11% |  |  |
| 3 – 4 | 144 | 61 | 83 | 31% | 29% |  |  |
| 5 – 6 | 59 | 27 | 32 | 14% | 11% |  |  |
| 7 – 8 | 145 | 66 | 79 | 33% | 28% |  |  |
| 9 – 12 | 37 | 18 | 19 | 9% | 7% |  |  |
| Missing | 42 | 0 | 42 |  |  |  |  |

^a^ Among 286 mothers enrolled in the baseline MARBLES, 35 mothers were excluded because they did not provide urine samples during 2^nd^ or 3^rd^ trimesters. Additional 43 mothers who miscarried or dropped out were excluded. We further restricted to children who received a final diagnosis using both Mullen Scales and ADOS scores (N=201).

^b^ P-value from the Pearson’s chi-squared test. Because of a large number of missing data in the baseline MARBLES participants, missing data were not included in this test.

^c^ Includes Black, Asian, and multiracial.

^d^ One of them has an identical twin sister with an autistic child, and the other mother has multiple siblings with autism. These two mothers were included in the current study because they have high-risk ASD genetic factors and exclusion of them did not change the results.

Note: Eleven mothers participated in the study for two different pregnancies and one mother participated for three different pregnancies over four years. Three pregnancies resulted in twins.

**Table S5.** Comparison between FMVs and 24-hour samples used as individual samples.

|  | **FMVs** (*n* = 355) | | **24-hour samples** (*n* = 82) | | p-value from *t* test |
| --- | --- | --- | --- | --- | --- |
|  | Median | (2.5^th^, 97.5^th^) | Median | (2.5^th^, 97.5^th^) |  |
| MEP | 20.84 | (4.19, 215.5) | 25.31 | (3.98, 271.7) | 0.74 |
| MiBP | 6.69 | (1.13, 28.9) | 7.18 | (2.05, 28.7) | 0.80 |
| MHiBP | 2.40 | (0.56, 9.74) | 2.51 | (0.66, 8.94) | 0.73 |
| MBP | 11.80 | (1.51, 51.8) | 12.54 | (3.60, 54.4) | 0.46 |
| MHBP | 1.00 | (0.25, 4.89) | 1.04 | (0.42, 4.31) | 0.69 |
| MBzP | 6.32 | (0.61, 48.3) | 5.74 | (1.20, 32.5) | 0.72 |
| MEHP | 2.40 | (0.52, 47.8) | 2.51 | (0.56, 38.9) | 1.00 |
| MEHHP | 10.50 | (1.4, 276.0) | 10.53 | (2.01, 154.0) | 0.54 |
| MEOHP | 8.60 | (1.23, 180.7) | 8.12 | (1.74, 106.4) | 0.62 |
| MECPP | 16.75 | (3.46, 386.3) | 15.62 | (4.99, 230.2) | 0.48 |
| MCPP | 1.38 | (0.39, 27.3) | 1.59 | (0.32, 7.16) | 0.16 |
| MNP | 1.00 | (0.36, 21.0) | 1.09 | (0.34, 6.56) | 0.26 |
| MCOP | 10.05 | (2.08, 339.2) | 9.65 | (1.28, 72.73) | 0.12 |
| MCNP | 2.40 | (0.6, 25.8) | 2.33 | (0.6, 26.1) | 0.67 |
| ∑DEHP | 0.13 | (0.02, 2.78) | 0.12 | (0.03, 1.77) | 0.56 |

**Table S6**. Comparison of phthalate metabolite concentrations in the current study to those in NHANES.

| Metabolites |  | Percent > LOD | Percentiles | | |  | Geometric mean (GM) | | |
| --- | --- | --- | --- | --- | --- | --- | --- | --- | --- |
|  | LOD  (µg/L) |  | 5^th^ | 50^th^ | 95^th^ |  | This study (2007-2014) | This study (2011-2012) ^a^ | NHANES (2011-2012) ^b^ |
| MEP | 1.2 | 100% | 5.5 | 22.0 | 193.5 |  | 25.3 | 22.8 | 37.7 |
| MiBP | 0.8 | 98% | 1.9 | 7.1 | 23.3 |  | 6.6 | 7.6 | 5.5 |
| MHiBP | 0.4 | 97% | 0.8 | 2.5 | 8.5 |  | 2.4 | 2.7 | NM |
| MBP | 0.4 | 99% | 3.4 | 12.1 | 40.9 |  | 11.1 | 12.9 | 7.1 |
| MHBP | 0.4 | 82% | <LOD | 1.1 | 3.7 |  | 1.0 | 1.0 | NM |
| MBzP | 0.3 | 99% | 1.2 | 6.2 | 33.4 |  | 6.0 | 6.6 | 4.3 |
| MEHP | 0.8 | 83% | <LOD | 2.5 | 25.7 |  | 2.7 | 1.7 | 1.2 |
| MEHHP | 0.4 | 100% | 2.5 | 11.3 | 105.9 |  | 11.6 | 8.4 | 7.2 |
| MEOHP | 0.2 | 100% | 2.0 | 9.1 | 79.1 |  | 9.0 | 6.7 | 4.7 |
| MECPP | 0.4 | 100% | 5.2 | 17.7 | 152.4 |  | 19.7 | 13.7 | 11.8 |
| MCPP | 0.4 | 92% | 0.5 | 1.7 | 13.1 |  | 1.7 | 2.0 | 2.6 |
| MNP | 0.9 | 50% | <LOD | 1.1 | 8.1 |  | 1.2 | 1.1 | NA |
| MCOP | 0.3 | 100% | 2.8 | 12.6 | 144.7 |  | 12.7 | 17.7 | 17.1 |
| MCNP | 0.2 | 100% | 0.8 | 2.6 | 22.9 |  | 2.8 | 2.6 | 3.0 |

**Abbreviations -** GM: geometric mean, NA: not available, NM: compound not measured, LOD: limit of detection, <LOD: below LOD

^a^ N = 63 first morning voids

^b^ NHANES: female only between 2011 and 2012.

**Table S7.** Sensitivity analyses I: RRR for ASD and Non-TD (versus TD) in relation to phthalate biomarker concentrations during mid- to late pregnancy.

|  | **ASD versus TD** | |  | **Non-TD versus TD** | |
| --- | --- | --- | --- | --- | --- |
|  | **RRR** | **95%CI** |  | **RRR** | **95%CI** |
| (1) *Analysis additionally adjusted for interpregnancy interval* | | | | | |
| MEP | 1.23 | (0.81, 1.86) |  | 1.37 | (1.00, 1.88)* |
| MiBP | 0.64 | (0.36, 1.13) |  | 1.20 | (0.72, 1.99) |
| MHiBP | 0.65 | (0.35, 1.21) |  | 1.12 | (0.64, 1.94) |
| MBP | 0.87 | (0.44, 1.68) |  | 1.49 | (0.86, 2.56) |
| MHBP | 0.91 | (0.43, 1.87) |  | 1.35 | (0.75, 2.38) |
| MBzP | 0.83 | (0.53, 1.30) |  | 1.30 | (0.88, 1.88) |
| MCPP | 0.70 | (0.46, 1.04) |  | 1.48 | (0.97, 2.25) |
| MCOP | 0.78 | (0.53, 1.12) |  | 1.33 | (0.89, 1.95) |
| MCNP | 0.97 | (0.62, 1.48) |  | 1.46 | (1.00, 2.12)* |
| ΣDEHP ^b^ | 0.73 | (0.41, 1.28) |  | 1.31 | (0.86, 2.00) |
| (2) *Analysis additionally adjusted for gestational age at birth* | | | | | |
| MEP | 1.20 | (0.78, 1.83) |  | 1.37 | (0.99, 1.88) |
| MiBP | 0.59 | (0.31, 1.07) |  | 1.15 | (0.68, 1.90) |
| MHiBP | 0.63 | (0.33, 1.16) |  | 1.08 | (0.62, 1.86) |
| MBP | 0.84 | (0.42, 1.65) |  | 1.45 | (0.83, 2.52) |
| MHBP | 0.90 | (0.43, 1.84) |  | 1.33 | (0.76, 2.33) |
| MBzP | 0.86 | (0.54, 1.34) |  | 1.31 | (0.90, 1.91) |
| MCPP | 0.70 | (0.46, 1.05) |  | 1.45 | (0.96, 2.17) |
| MCOP | 0.76 | (0.52, 1.10) |  | 1.30 | (0.89, 1.89) |
| MCNP | 0.90 | (0.58, 1.39) |  | 1.38 | (0.96, 1.97) |
| ΣDEHP ^b^ | 0.71 | (0.41, 1.21) |  | 1.33 | (0.86, 2.05) |
| (3) *Analysis additionally adjusted for maternal age at delivery* | | | | | |
| MEP | 1.27 | (0.84, 1.89) |  | 1.40 | (1.02, 1.92)* |
| MiBP | 0.66 | (0.36, 1.16) |  | 1.20 | (0.72, 1.98) |
| MHiBP | 0.66 | (0.34, 1.24) |  | 1.12 | (0.64, 1.93) |
| MBP | 0.87 | (0.44, 1.73) |  | 1.49 | (0.86, 2.56) |
| MHBP | 0.96 | (0.45, 2.00) |  | 1.38 | (0.77, 2.43) |
| MBzP | 0.83 | (0.52, 1.31) |  | 1.31 | (0.89, 1.89) |
| MCPP | 0.67 | (0.43, 1.01) |  | 1.42 | (0.94, 2.14) |
| MCOP | 0.74 | (0.50, 1.07) |  | 1.28 | (0.88, 1.86) |
| MCNP | 0.89 | (0.59, 1.32) |  | 1.38 | (0.95, 1.98) |
| ΣDEHP ^b^ | 0.70 | (0.38, 1.26) |  | 1.36 | (0.88, 2.08) |
|  |  |  |  |  |  |
| (4) *Analysis additionally adjusted for child’s race/ethnicity* | | | | | |
| MEP | 1.20 | (0.78, 1.83) |  | 1.37 | (0.99, 1.88) |
| MiBP | 0.64 | (0.36, 1.10) |  | 1.17 | (0.70, 1.92) |
| MHiBP | 0.65 | (0.35, 1.18) |  | 1.10 | (0.63, 1.89) |
| MBP | 0.87 | (0.44, 1.68) |  | 1.47 | (0.84, 2.53) |
| MHBP | 0.91 | (0.43, 1.87) |  | 1.34 | (0.75, 2.37) |
| MBzP | 0.85 | (0.54, 1.34) |  | 1.33 | (0.91, 1.93) |
| MCPP | 0.72 | (0.47, 1.08) |  | 1.45 | (0.97, 2.15) |
| MCOP | 0.77 | (0.53, 1.12) |  | 1.29 | (0.89, 1.87) |
| MCNP | 0.93 | (0.62, 1.39) |  | 1.38 | (0.96, 1.99) |
| ΣDEHP ^b^ | 0.78 | (0.44, 1.37) |  | 1.40 | (0.90, 2.17) |

^a^ Adjusted for pre-pregnancy BMI, year of birth (linear and squared terms), and homeownership

^b^ Because correlations among DEHP metabolites were high (*r* = 0.84 - 0.99), the molar sum of

DEHP metabolites was only examined (ΣDEHP, µmol/L).

* p-value <0.05, # p-value <0.10

Note: MNP was excluded from this table because approximately 50% of samples had detectable concentrations.

Abbreviation: monoethyl phthalate (MEP), mono-isobutyl phthalate (MiBP), monohydroxy-isobutyl phthalate (MHiBP), mono-n-butyl phthalate (MBP), monohydroxy-n-butyl phthalate (MHBP), monobenzyl phthalate (MBzP), mono(2-ethylhexyl) phthalate (MEHP), mono(2-ethyl-5-hydroxyhexyl) phthalate (MEHHP), mono(2-ethyl-5-oxohexyl) phthalate (MEOHP), mono(2-ethyl-5-carboxypentyl) phthalate (MECPP), mono(3-carboxypropyl) phthalate (MCPP), mono-isononyl phthalate (MNP), mono-carboxyisooctyl phthalate (MCOP), and mono-carboxyisononyl phthalate (MCNP), the molar sum (µmol/L) of DEHP metabolites (ΣDEHP = MEHP + MEHHP + MEOHP + MECPP)

**Table S8:** Sensitivity analyses II: RRR for ASD and Non-TD (versus TD) in relation to phthalate biomarker concentrations during mid- to late pregnancy.

|  | **ASD versus TD** | |  | **Non-TD versus TD** | |
| --- | --- | --- | --- | --- | --- |
|  | **RRR** | **95%CI** |  | **RRR** | **95%CI** |
| (1) *Exclusion of the top 2.5 percentiles of phthalate metabolite concentrations* | | | | | |
| MEP | 1.02 | (0.65, 1.58) |  | 1.29 | (0.87, 1.89) |
| MiBP | 0.71 | (0.38, 1.31) |  | 1.23 | (0.73, 2.07) |
| MHiBP | 0.62 | (0.31, 1.22) |  | 1.04 | (0.58, 1.87) |
| MBP | 0.62 | (0.32, 1.17) |  | 1.47 | (0.78, 2.76) |
| MHBP | 0.81 | (0.37, 1.74) |  | 1.35 | (0.70, 2.59) |
| MBzP | 0.81 | (0.50, 1.31) |  | 1.32 | (0.87, 1.99) |
| MCPP | 0.78 | (0.50, 1.21) |  | 1.65 | (1.00, 2.71)* |
| MCOP | 0.82 | (0.54, 1.23) |  | 1.35 | (0.88, 2.05) |
| MCNP | 0.99 | (0.60, 1.63) |  | 1.27 | (0.80, 1.99) |
| ΣDEHP ^b^ | 0.82 | (0.45, 1.49) |  | 1.58 | (0.96, 2.56) |
| (2) *Exclusion of 6 twins* (*n* = 195) | | | | | |
| MEP | 1.23 | (0.80, 1.87) |  | 1.34 | (0.96, 1.84) |
| MiBP | 0.58 | (0.31, 1.05) |  | 1.07 | (0.63, 1.78) |
| MHiBP | 0.61 | (0.32, 1.14) |  | 1.04 | (0.60, 1.78) |
| MBP | 0.85 | (0.42, 1.67) |  | 1.38 | (0.80, 2.39) |
| MHBP | 0.90 | (0.43, 1.86) |  | 1.32 | (0.75, 2.29) |
| MBzP | 0.83 | (0.52, 1.30) |  | 1.30 | (0.89, 1.87) |
| MCPP | 0.73 | (0.48, 1.09) |  | 1.47 | (0.97, 2.22) |
| MCOP | 0.79 | (0.54, 1.15) |  | 1.33 | (0.91, 1.93) |
| MCNP | 0.94 | (0.62, 1.41) |  | 1.36 | (0.94, 1.94) |
| ΣDEHP ^b^ | 0.78 | (0.44, 1.36) |  | 1.36 | (0.88, 2.08) |
| (3) *Adjust the varying number of urine samples per mother* | | | | |  |
| MEP | 1.25 | (0.82, 1.89) |  | 1.35 | (0.97, 1.87) |
| MiBP | 0.61 | (0.34, 1.07) |  | 1.16 | (0.70, 1.90) |
| MHiBP | 0.61 | (0.33, 1.14) |  | 1.09 | (0.63, 1.88) |
| MBP | 0.90 | (0.45, 1.76) |  | 1.42 | (0.81, 2.46) |
| MHBP | 0.92 | (0.45, 1.87) |  | 1.33 | (0.74, 2.35) |
| MBzP | 0.84 | (0.53, 1.30) |  | 1.31 | (0.89, 1.89) |
| MCPP | 0.67 | (0.43, 1.04) |  | 1.43 | (0.95, 2.14) |
| MCOP | 0.72 | (0.47, 1.07) |  | 1.27 | (0.87, 1.82) |
| MCNP | 0.93 | (0.61, 1.40) |  | 1.38 | (0.94, 1.99) |
| ΣDEHP ^b^ | 0.81 | (0.44, 1.49) |  | 1.42 | (0.90, 2.22) |

^a^ Adjusted for pre-pregnancy BMI, year of birth (linear and squared terms), and homeownership

^b^ Because correlations among DEHP metabolites were high (*r* = 0.84 - 0.99), the molar sum of

DEHP metabolites was only examined (ΣDEHP, µmol/L).

* p-value <0.05, # p-value <0.10

Note: MNP was excluded from this table because approximately 50% of samples had detectable concentrations. See Table S3 for abbreviations.

Table S9. Stratified analyses by prenatal vitamin use during the first month of pregnancy using samples during mid-pregnancy.

|  | Prenatal vitamin use (*n* = 73; TD = 40, ASD = 11, Non-TD = 22) | |  | No prenatal vitamin use (*n* = 57; TD = 22, ASD = 17, Non-TD = 18) | |  | P-value for interaction ^c^ |
| --- | --- | --- | --- | --- | --- | --- | --- |
|  | RRR ^a^ | 95% CI |  | RRR ^a^ | 95% CI |  |  |
| *ASD versus TD* | | | | | | | |
| MEP | 1.05 | (0.44, 2.53) |  | 0.77 | (0.40, 1.49) |  | 0.57 |
| MiBP | 0.15 | (0.05, 0.44)* |  | 1.02 | (0.37, 2.76) |  | 0.03 |
| MHiBP | 0.23 | (0.08, 0.68)* |  | 1.04 | (0.36, 3.02) |  | 0.13 |
| MBP | 0.22 | (0.04, 1.25) |  | 0.91 | (0.29, 2.88) |  | 0.11 |
| MHBP | 0.29 | (0.08, 1.13) |  | 1.04 | (0.34, 3.15) |  | 0.16 |
| MBzP | 0.67 | (0.28, 1.59) |  | 0.92 | (0.36, 2.34) |  | 0.51 |
| MCPP | 0.08 | (0.02, 0.42)* |  | 0.95 | (0.45, 2.00) |  | 0.00 |
| MCOP | 0.41 | (0.21, 0.79)* |  | 0.94 | (0.52, 1.70) |  | 0.08 |
| MCNP | 0.75 | (0.40, 1.41) |  | 0.93 | (0.39, 2.22) |  | 0.61 |
| ΣDEHP ^b^ | 0.49 | (0.18, 1.35) |  | 1.09 | (0.45, 2.68) |  | 0.19 |
| *Non-TD versus TD* | | | | | | | |
| MEP | 1.32 | (0.74, 2.35) |  | 1.33 | (0.66, 2.69) |  | 0.87 |
| MiBP | 0.61 | (0.19, 1.97) |  | 0.98 | (0.33, 2.88) |  | 0.43 |
| MHiBP | 0.54 | (0.14, 2.07) |  | 0.91 | (0.27, 3.04) |  | 0.38 |
| MBP | 0.75 | (0.26, 2.20) |  | 1.06 | (0.34, 3.24) |  | 0.40 |
| MHBP | 0.69 | (0.25, 1.95) |  | 1.42 | (0.46, 4.42) |  | 0.25 |
| MBzP | 1.38 | (0.68, 2.80) |  | 1.17 | (0.49, 2.76) |  | 0.79 |
| MCPP | 0.99 | (0.48, 2.02) |  | 2.32 | (0.99, 5.43) |  | 0.07 |
| MCOP | 0.91 | (0.44, 1.89) |  | 1.64 | (0.84, 3.20) |  | 0.23 |
| MCNP | 0.70 | (0.32, 1.54) |  | 2.73 | (1.20, 6.21)* |  | 0.01 |
| ΣDEHP ^b^ | 1.06 | (0.60, 1.89) |  | 1.34 | (0.60, 3.01) |  | 0.27 |

^a^ Adjusted for pre-pregnancy BMI, year of birth (linear and squared terms), and homeownership.

^b^ Due to high correlations among DEHP metabolites (*r* = 0.84 - 0.99), the molar sum of

DEHP metabolites was only examined (ΣDEHP, µmol/L).

^c^ P-value for interaction from the separate regression models.

* p-value <0.05

Table S10. Stratified analyses by prenatal vitamin use during the first month of pregnancy using samples during late-pregnancy.

|  | Prenatal vitamin use (*n* = 103; TD = 60, ASD = 15, Non-TD = 28) | |  | No prenatal vitamin use (*n* = 78; TD = 35, ASD = 23, Non-TD = 20) | |  | P-value for interaction ^c^ |
| --- | --- | --- | --- | --- | --- | --- | --- |
|  | RRR ^a^ | 95% CI |  | RRR ^a^ | 95% CI |  |  |
| *ASD versus TD* | | | | | | | |
| MEP | 1.71 | (0.71, 4.13) |  | 0.87 | (0.47, 1.60) |  | 0.23 |
| MiBP | 0.48 | (0.22, 1.04) |  | 0.62 | (0.28, 1.37) |  | 0.82 |
| MHiBP | 0.55 | (0.23, 1.35) |  | 0.51 | (0.22, 1.16) |  | 0.63 |
| MBP | 0.66 | (0.23, 1.89) |  | 1.21 | (0.45, 3.29) |  | 0.37 |
| MHBP | 0.97 | (0.34, 2.78) |  | 1.25 | (0.48, 3.23) |  | 0.59 |
| MBzP | 0.96 | (0.46, 2.00) |  | 0.81 | (0.47, 1.40) |  | 0.99 |
| MCPP | 0.61 | (0.32, 1.14) |  | 1.45 | (0.73, 2.90) |  | 0.08 |
| MCOP | 0.60 | (0.34, 1.07) |  | 1.32 | (0.80, 2.16) |  | 0.07 |
| MCNP | 0.85 | (0.48, 1.51) |  | 1.10 | (0.55, 2.20) |  | 0.58 |
| ΣDEHP ^b^ | 0.67 | (0.23, 1.93) |  | 1.22 | (0.49, 3.06) |  | 0.25 |
| *Non-TD versus TD* | | | | | | | |
| MEP | 1.53 | (1.01, 2.33)* |  | 1.07 | (0.61, 1.87) |  | 0.38 |
| MiBP | 1.43 | (0.65, 3.12) |  | 1.16 | (0.59, 2.29) |  | 0.72 |
| MHiBP | 1.40 | (0.55, 3.58) |  | 0.99 | (0.49, 2.03) |  | 0.59 |
| MBP | 1.72 | (0.84, 3.55) |  | 1.42 | (0.65, 3.14) |  | 0.83 |
| MHBP | 1.72 | (0.79, 3.77) |  | 1.50 | (0.57, 4.00) |  | 0.72 |
| MBzP | 1.65 | (0.96, 2.83) |  | 1.11 | (0.62, 1.98) |  | 0.43 |
| MCPP | 0.96 | (0.59, 1.55) |  | 2.07 | (1.00, 4.31)* |  | 0.07 |
| MCOP | 0.91 | (0.59, 1.40) |  | 1.21 | (0.64, 2.30) |  | 0.54 |
| MCNP | 0.89 | (0.55, 1.44) |  | 2.17 | (1.12, 4.23)* |  | 0.03 |
| ΣDEHP ^b^ | 1.22 | (0.72, 2.08) |  | 1.54 | (0.79, 3.00) |  | 0.59 |

^a^ Adjusted for pre-pregnancy BMI, year of birth (linear and squared terms), and homeownership.

^b^ Due to high correlations among DEHP metabolites (*r* = 0.84 - 0.99), the molar sum of

DEHP metabolites was only examined (ΣDEHP, µmol/L).

^c^ P-value for interaction from the separate regression models.

* p-value <0.05

Table S11. Stratified analyses by sex using samples during mid-pregnancy.

|  | Girls (*n* = 47; TD = 28, ASD = 5, Non-TD = 14) | |  | Boys (*n* = 85; TD = 34, ASD = 24, Non-TD = 27) | |  | P-value for interaction ^c^ |
| --- | --- | --- | --- | --- | --- | --- | --- |
|  | RRR ^a^ | 95% CI |  | RRR ^a^ | 95% CI |  |  |
| *ASD versus TD* | | | | | | | |
| MEP | 0.92 | (0.47, 1.82) |  | 1.03 | (0.58, 1.84) |  | 0.62 |
| MiBP | 4.14 | (0.49, 34.6) |  | 0.29 | (0.10, 0.82)* |  | 0.01 |
| MHiBP | 3.38 | (0.43, 26.4) |  | 0.31 | (0.12, 0.77)* |  | 0.03 |
| MBP | 2.41 | (0.13, 45.7) |  | 0.41 | (0.15, 1.08) |  | 0.22 |
| MHBP | 0.97 | (0.13, 7.53) |  | 0.51 | (0.20, 1.26) |  | 0.45 |
| MBzP | 0.58 | (0.17, 1.94) |  | 0.90 | (0.46, 1.79) |  | 0.65 |
| MCPP | 0.86 | (0.30, 2.47) |  | 0.37 | (0.17, 0.82)* |  | 0.12 |
| MCOP | 0.77 | (0.32, 1.89) |  | 0.64 | (0.38, 1.08) |  | 0.47 |
| MCNP | 0.79 | (0.35, 1.78) |  | 0.85 | (0.39, 1.88) |  | 0.82 |
| ΣDEHP ^b^ | 0.64 | (0.16, 2.57) |  | 0.94 | (0.51, 1.72) |  | 0.22 |
| *Non-TD versus TD* | | | | | | | |
| MEP | 0.74 | (0.31, 1.77) |  | 2.19 | (1.02, 4.70)* |  | 0.05 |
| MiBP | 0.66 | (0.14, 3.07) |  | 0.73 | (0.28, 1.86) |  | 0.89 |
| MHiBP | 1.00 | (0.24, 4.07) |  | 0.61 | (0.23, 1.67) |  | 0.85 |
| MBP | 0.31 | (0.08, 1.25) |  | 1.04 | (0.43, 2.52) |  | 0.22 |
| MHBP | 0.38 | (0.13, 1.15) |  | 1.13 | (0.47, 2.73) |  | 0.12 |
| MBzP | 0.71 | (0.28, 1.77) |  | 1.78 | (0.94, 3.38) |  | 0.24 |
| MCPP | 0.93 | (0.40, 2.17) |  | 1.62 | (0.86, 3.07) |  | 0.39 |
| MCOP | 1.14 | (0.56, 2.30) |  | 1.17 | (0.61, 2.24) |  | 0.72 |
| MCNP | 0.87 | (0.48, 1.57) |  | 1.70 | (0.74, 3.91) |  | 0.29 |
| ΣDEHP ^b^ | 0.72 | (0.26, 1.99) |  | 1.43 | (0.77, 2.65) |  | 0.28 |

^a^ Adjusted for pre-pregnancy BMI, year of birth (linear and squared terms), and homeownership.

^b^ Due to high correlations among DEHP metabolites (*r* = 0.84 - 0.99), the molar sum of

DEHP metabolites was only examined (ΣDEHP, µmol/L).

^c^ P-value for interaction from the separate regression models.

* p-value <0.05

Table S12. Stratified analyses by sex using samples during late-pregnancy.

|  | Girls (*n* = 76; TD = 44, ASD = 12, Non-TD = 20) | |  | Boys (*n* = 113; TD = 52, ASD = 31, Non-TD = 30) | |  | P-value for interaction ^c^ |
| --- | --- | --- | --- | --- | --- | --- | --- |
|  | RRR ^a^ | 95% CI |  | RRR ^a^ | 95% CI |  |  |
| *ASD versus TD* | | | | | | | |
| MEP | 0.92 | (0.47, 1.82) |  | 1.56 | (0.96, 2.56) |  | 0.02 |
| MiBP | 4.14 | (0.49, 34.6) |  | 0.50 | (0.23, 1.07) |  | 0.54 |
| MHiBP | 3.38 | (0.43, 26.4) |  | 0.54 | (0.26, 1.11) |  | 0.75 |
| MBP | 2.41 | (0.13, 45.7) |  | 1.19 | (0.52, 2.74) |  | 0.89 |
| MHBP | 0.97 | (0.13, 7.53) |  | 1.51 | (0.71, 3.24) |  | 0.78 |
| MBzP | 0.58 | (0.17, 1.94) |  | 1.17 | (0.69, 1.99) |  | 0.15 |
| MCPP | 0.86 | (0.30, 2.47) |  | 1.16 | (0.64, 2.09) |  | 0.38 |
| MCOP | 0.77 | (0.32, 1.89) |  | 0.91 | (0.56, 1.48) |  | 0.70 |
| MCNP | 0.79 | (0.35, 1.78) |  | 1.21 | (0.61, 2.38) |  | 0.27 |
| ΣDEHP ^b^ | 0.64 | (0.16, 2.57) |  | 0.91 | (0.43, 1.91) |  | 0.51 |
| *Non-TD versus TD* | | | | | | | |
| MEP | 0.74 | (0.31, 1.77) |  | 1.38 | (0.91, 2.08) |  | 0.36 |
| MiBP | 0.66 | (0.14, 3.07) |  | 1.18 | (0.59, 2.38) |  | 0.92 |
| MHiBP | 1.00 | (0.24, 4.07) |  | 0.98 | (0.48, 2.01) |  | 0.52 |
| MBP | 0.31 | (0.08, 1.25) |  | 1.86 | (0.93, 3.71) |  | 0.63 |
| MHBP | 0.38 | (0.13, 1.15) |  | 1.82 | (0.92, 3.57) |  | 0.57 |
| MBzP | 0.71 | (0.28, 1.77) |  | 1.86 | (1.13, 3.06)* |  | 0.07 |
| MCPP | 0.93 | (0.40, 2.17) |  | 1.51 | (0.83, 2.76) |  | 0.27 |
| MCOP | 1.14 | (0.56, 2.30) |  | 1.10 | (0.70, 1.71) |  | 0.56 |
| MCNP | 0.87 | (0.48, 1.57) |  | 1.34 | (0.80, 2.25) |  | 0.55 |
| ΣDEHP ^b^ | 0.72 | (0.26, 1.99) |  | 1.62 | (0.89, 2.95) |  | 0.27 |

^a^ Adjusted for pre-pregnancy BMI, year of birth (linear and squared terms), and homeownership.

^b^ Due to high correlations among DEHP metabolites (*r* = 0.84 - 0.99), the molar sum of

DEHP metabolites was only examined (ΣDEHP, µmol/L).

^c^ P-value for interaction from the separate regression models.

* p-value <0.05

**Table S13**. Spearman’s correlations of average phthalate metabolite concentrations between the 2^nd^ trimester samples (n =134) and the 3^rd^ trimester samples (n =190).

| Metabolites* | Spearman’s correlation coefficient |
| --- | --- |
| MEP | 0.62 |
| MiBP | 0.66 |
| MHiBP | 0.66 |
| MBP | 0.58 |
| MHBP | 0.60 |
| MBzP | 0.83 |
| MEHP | 0.44 |
| MEHHP | 0.42 |
| MEOHP | 0.46 |
| MECPP | 0.42 |
| MCPP | 0.07 |
| MCOP | 0.11 |
| MCNP | 0.14 |

*MNP was excluded from this table because approximately 50% of samples had detectable concentrations.

Figure S1. Selection of mother-child pairs used in this study.

Figure S2. Type and number of urine samples analyzed in this study.


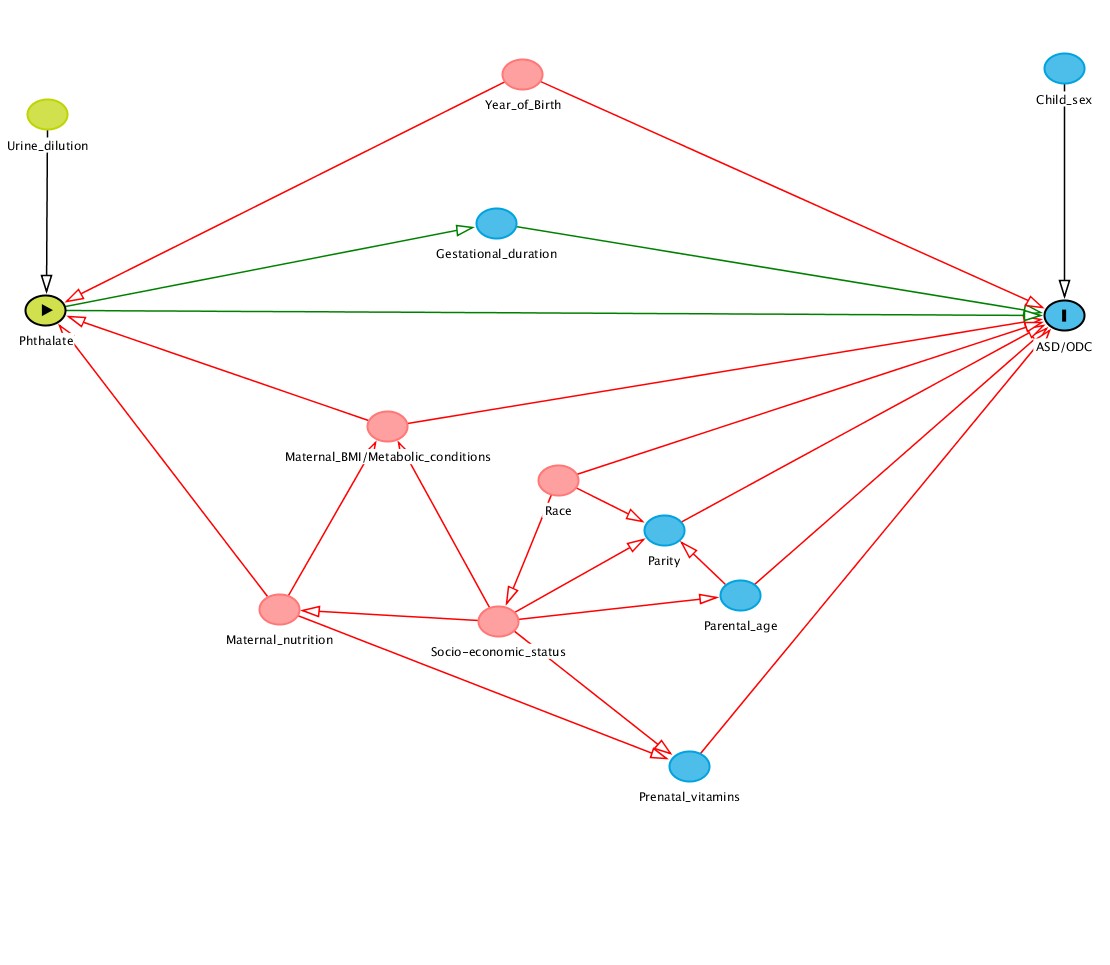


Figure S3. Directed acyclic graph used to identify and select adjustment factors. Green circles represent ancestors of the exposure, blue circles ancestors of the outcome, pink circles ancestors of both exposure and outcome (Textor et al. 2011).


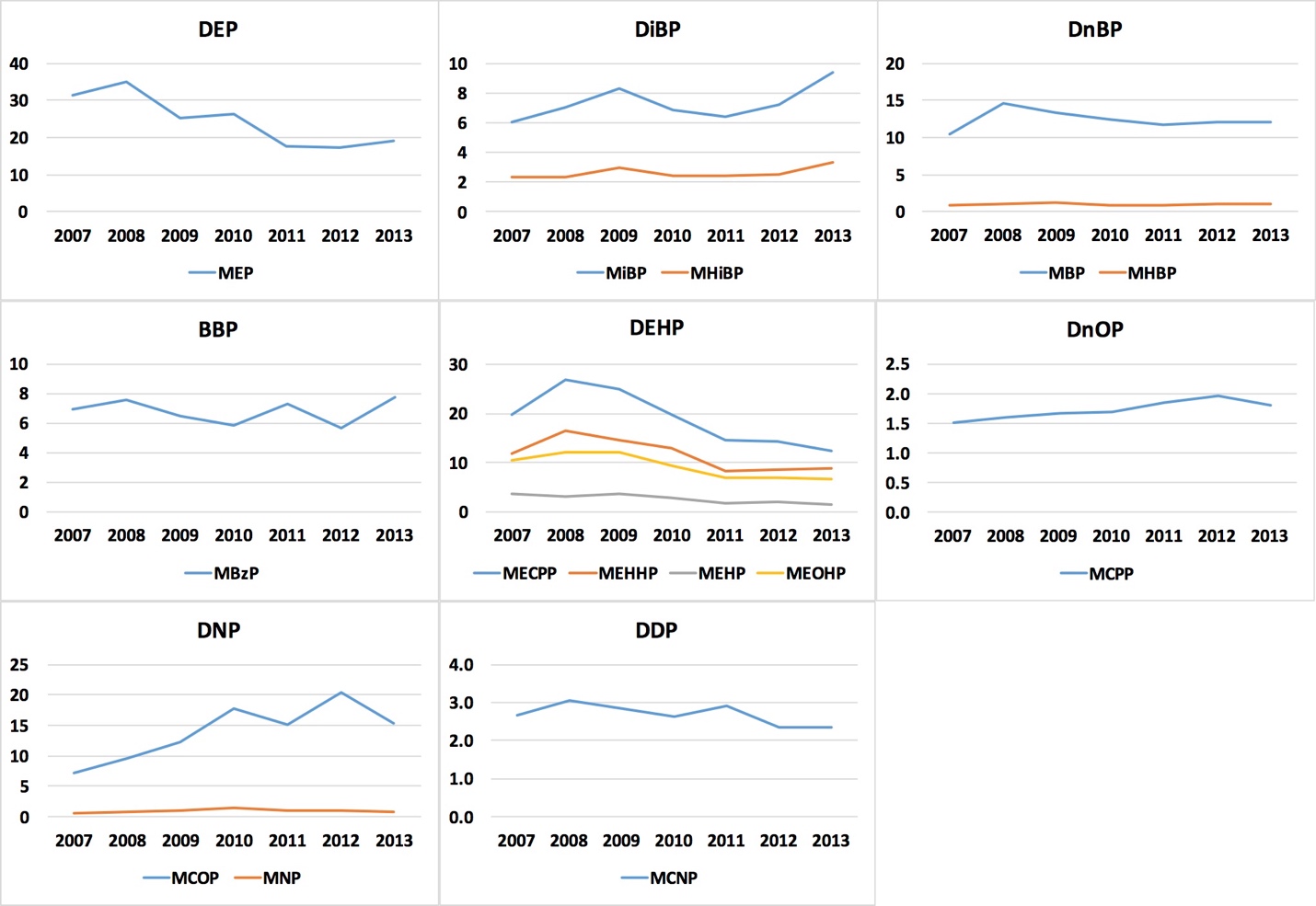


Figure S4. Temporal trends of median phthalate biomarker concentrations (µg/L) in the selected MARBLES participants. Each line represents median concentrations of a metabolite.

Figure S5. ICC and 95% CI of ln-transformed phthalate metabolite concentrations using samples collected during each of the 2^nd^ and 3^rd^ trimesters: (1) two first FMVs (*n* = 204 from 102 mothers) and (2) two pooled samples (*n* = 118 from 59 mothers).

**References**

Textor J, Hardt J, Knuppel S. 2011. Dagitty: A graphical tool for analyzing causal diagrams. Epidemiology (Cambridge, Mass) 22:745.
